# Marburg virus glycoprotein mRNA vaccine is more protective than a virus-like particle-forming mRNA vaccine

**DOI:** 10.1172/JCI194586

**Published:** 2025-07-03

**Authors:** Chandru Subramani, Michelle Meyer, Matthew A. Hyde, Margaret E. Comeaux, Haiping Hao, James E. Crowe, Vsevolod L. Popov, Harshwardhan Thaker, Sunny Himansu, Andrea Carfi, Alexander Bukreyev

**Affiliations:** 1 Department of Pathology, University of Texas Medical Branch (UTMB) at Galveston, Galveston, Texas, USA.; 2 Galveston National Laboratory, Galveston, Texas, USA.; 3 Animal Resources Center and; 4 Department of Biochemistry and Molecular Biology, UTMB, Galveston, Texas, USA.; 5 Vanderbilt Vaccine Center,; 6 Department of Pathology, Microbiology, and Immunology, and; 7 Department of Pediatrics (Infectious Diseases), Vanderbilt University Medical Center, Nashville, Tennessee, USA.; 8 Moderna Inc., Cambridge, Massachusetts, USA.; 9 Department of Microbiology and Immunology and; 10 Center for Biodefense and Emerging Viral Infections, UTMB, Galveston, Texas, USA.

**Keywords:** Infectious disease, Virology, Cellular immune response, Immunoglobulins, Vaccines

## Abstract

Although virus-like particle (VLP) vaccines were shown to be effective against several viruses, their advantage over vaccines that include envelope protein only is not completely clear, particularly for mRNA-encoded VLPs. We conducted a side-by-side comparison of the immunogenicity and protective efficacy of mRNA vaccines encoding the Marburg virus (MARV) full-length glycoprotein (GP) delivered alone or as a VLP. Electron microscopy confirmed VLP formation when MARV GP and matrix protein VP40 were coexpressed. We vaccinated guinea pigs with a 2-component mRNA vaccine encoding GP and VP40 (VLP) or GP alone. At the highest dose, both vaccines protected fully, although the VLP vaccine elicited a slightly lower humoral response than did the GP-only mRNA vaccine. However, at low doses, GP-only mRNA conferred 100% protection, whereas the VLP vaccine conferred only partial protection. In mice, VLP mRNA induced a moderate preference for GP-specific CD8^+^ T cell responses, whereas the GP-only mRNA somewhat favored CD4^+^ T cell responses. Guinea pig whole-blood RNA-Seq revealed that the VLP vaccine downregulated genes associated with various biological and metabolic processes, including the NF-κB signaling pathway, whereas the GP-only vaccine upregulated IFN signaling. Overall, the VLP mRNA vaccine was less immunogenic and protective, whereas the GP-only mRNA vaccine conferred robust protection with a dose of as little as 1 μg in guinea pigs.

## Abstract 2

mRNA platform was used to develop vaccines against Marburg virus as either glycoprotein-expressing mRNA, or glycoprotein and matrix VP40, resulting in formation of virus-like partielces.

## Graphical abstract

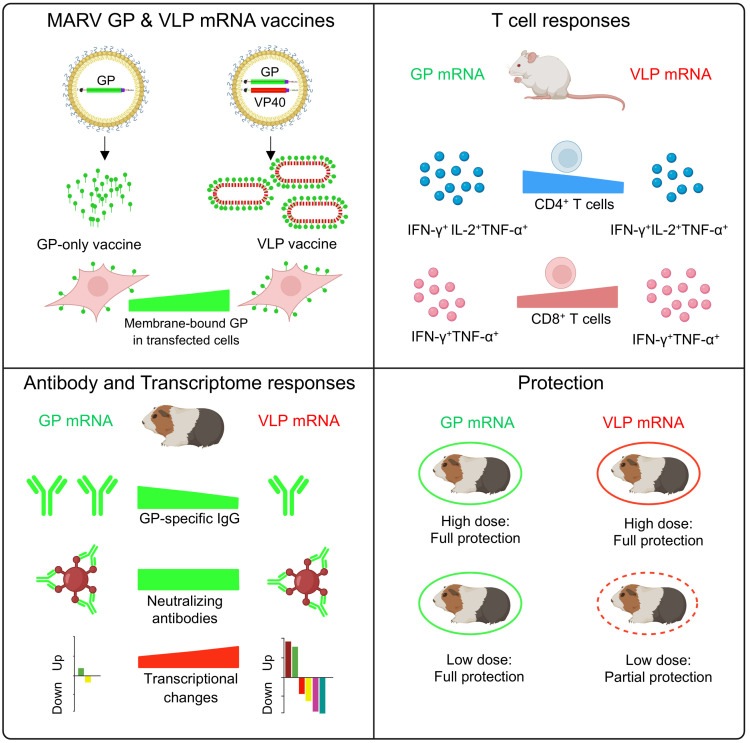

## Introduction

Marburg virus (MARV) causes a severe disease in humans, characterized by the onset of fever and headache, and can lead to hemorrhagic manifestations with severe bleeding and multiorgan failure, with up to 88% mortality (1). MARV is a member of the *Filoviridae* family, which includes the highly pathogenic viruses Ebola, Sudan, Bungibugyo, and Taï Forest. Two nonendemic countries, Equatorial Guinea and Tanzania, experienced their first-ever outbreak simultaneously in 2023 (2), and new outbreaks occurred in Rwanda in September–November 2024 (3, 4) and in Tanzania in January 2025 (5), highlighting the threat of major outbreaks. There is no approved vaccine against MARV. The virus has a 19.1 kb single-stranded negative-sense RNA genome that encodes 7 structural proteins. The glycoprotein (GP) is the sole envelope protein that mediates virus attachment and entry into host cells (6). GP is a key target of antibodies such as MR186 and MR191, shown to be protective in nonhuman primates (NHPs) (7, 8). Consequently, MARV vaccine development has primarily focused on GP as the immunogen.

Several vaccine platforms, including DNA (9), protein (10), and viral vectors such as adenovirus (Ad) (11), vesicular stomatitis virus (VSV) (12), and modified vaccinia Ankara (MVA) (13), were used for the development of monovalent GP–based vaccines against MARV. Preclinical studies also reported multivalent vaccines targeting Ebola, Sudan, Lassa, and Marburg viruses in various combinations (14–16). The chimpanzee adenovirus 3 (cAd3) Marburg GP vaccine induced GP-binding IgG antibodies in 95% of participants and significant GP-specific T cell responses in phase I clinical trial (17). The Ad26.Filo and MVA-BN-Filo multivalent vaccine showed encouraging results in phase 1 trial, but the MARV-specific antibody response was not assessed in the subsequent trials (18–20).

Apart from MARV vaccines solely delivering GP, we and others have reported preclinical studies on MARV-like particle (virus-like particle [VLP]) vaccines, which in addition to GP, include the matrix protein VP40 with or without the nucleoprotein (NP) (13, 21). A growing body of literature supports the view that VLPs are potent inducers of the immune response by virtue of their structural features, which display highly ordered and repetitive structures that closely resemble the actual virus particles (22). Additionally, the antigenic conformation of viral proteins is retained in VLPs. Most nonenveloped viral capsids self-assemble to form VLPs without the help of accessory viral proteins (23). All 3 licensed VLP vaccines against papillomavirus, hepatitis B, and hepatitis E are composed of a single protective antibody–inducing antigen (23). However, the external envelope protein of most enveloped viruses requires internal structural proteins, typically the matrix and nucleocapsid proteins, to form VLPs. Hence, VLP vaccines of enveloped viruses carry accessory viral antigens that can induce non-neutralizing antibodies and possibly protective T cell responses. These viral accessory antigens may influence immune responses by cooperating or competing with the epitopes of the envelope protein(s) (24, 25). Nonenvelope components could also enhance the protection conferred by vaccines based on the envelope protein alone due to formation of VLPs, which are typically highly immunogenic (22). Therefore, it is expected that any immune response induced by the accessory internal protein required for the formation of VLPs should contribute the protection or, at a minimum, not reduce the envelope-mediated protective response.

MARV and the other members of the *Filoviridae* family, including the Ebola virus, require VP40 for VLP production (26, 27). While the presence of VP40 is an absolute requirement for filovirus VLP formation, some studies have included the NP component in the VLP vaccine. A MARV protein–based VLP vaccine composed of GP, VP40, and NP showed 100% protection in NHPs (21). In addition, we reported that MVA vectors expressing GP and VP40, the minimum components required for filovirus VLPs, provide complete protection against MARV or Sudan virus in guinea pigs (13, 28). The success of mRNA vaccines in combating the SARS-CoV-2 pandemic (29, 30) has accelerated the development of mRNA vaccines against an array of viruses such as HIV-1 (31), influenza virus (32), respiratory syncytial virus (33), Andes virus (ANDV) (34), Lassa virus (35), and Ebola virus (36), demonstrating the versatility and adaptability of this new vaccine platform. Moreover, 2 studies have reported VLP mRNA vaccines for HIV-1 (31) and SARS-CoV-2 (37). However, it is unclear if the advantages of a VLP-based vaccine platform can be generalized.

**Figure 1:**
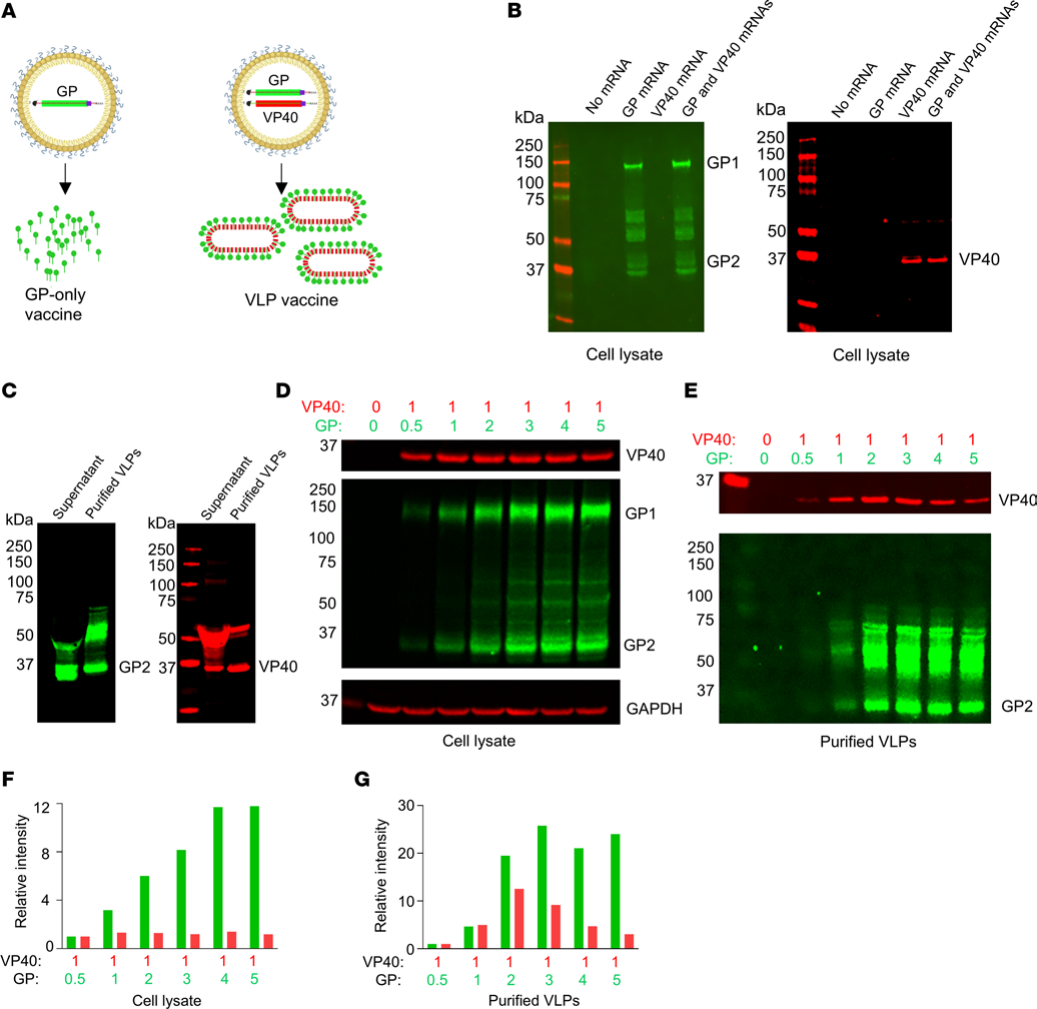
Optimization of MARV VLP generation from GP and VP40 mRNA in HEK293T cells. (**A**) Schematic of MARV GP and VLP mRNA LNP vaccines. Schematic was created with BioRender. (**B**) Immunoblots of GP and VP40 mRNA–transfected HEK293T cell lysates. (**C**) Immunoblots of GP and VP40 for the sucrose cushion concentrated VLPs. (**D** and **E**) The indicated concentrations of GP mRNA (μg) were transfected with VP40 mRNA in HEK293T cells to determine the optimal ratio for VLP generation. Immunoblots of cell lysates (**D**) and VLPs (**E**). GAPDH was used as a loading control. (**F** and **G**) Comparison of the intensity of GP and VP40 bands in the blots in **D** and **E**. Molecular weights in kDa are shown at the left.

This study aimed to develop a MARV methyl-pseudouridine–modified mRNA vaccine encoding the minimal components for VLP formation and compare its immunogenicity and protective efficacy with a GP-only mRNA vaccine (Figure 1A). We used a MARV-lethal guinea pig model to show that the GP mRNA vaccine was highly immunogenic and fully protective against MARV infection at a dose as low as 1 μg, whereas the VLP-based vaccine was less immunogenic and protective. We demonstrated that GP mRNA induced moderately elevated CD4^+^ T cell responses in mice, while VLP mRNA induced moderately increased GP-specific CD8^+^ T cell responses. The GP mRNA also induced minimal changes in the guinea pig blood transcriptome after vaccination, while the VLP mRNA downregulated genes associated with cellular and metabolic processes and the NF-κB signaling pathway. These findings shed light on differential immune responses induced by GP- and VLP-based mRNA vaccines, which is important for the development of effective vaccines and mitigation of their adverse effects.

## Results

### MARV GP and VP40 mRNA coexpression generates virus-like particles.

Several studies have demonstrated that MARV GP and VP40 coexpression generates VLPs (13, 26). Here, we tested the ability of MARV GP and VP40 mRNA coexpression to produce VLPs. To verify the expression of GP and VP40, HEK293T cells were individually transfected or cotransfected with mRNA for 24 hours. Western blotting of cell lysates (13, 38) revealed the expected molecular weight of GP and VP40 proteins (Figure 1B). To confirm that the VLPs were secreted, supernatants collected 48 hours after transfection of HEK293T cells were clarified by centrifugation, and VLPs were concentrated by ultracentrifugation and then analyzed by Western blotting (Figure 1C).

To determine the optimal GP/VP40 mRNA ratio that promotes high VLP yields and incorporation of GP, we cotransfected GP mRNA in a range of concentrations (0.5–5 μg) with 1 μg VP40 mRNA. Intracellular GP levels increased in a GP mRNA concentration–dependent manner, plateauing at 4 μg, while VP40 intracellular levels remained constant. However, the highest incorporation of VP40 into VLPs was achieved at the 1:2 ratio of VP40/GP mRNA, which subsequently decreased as the GP mRNA concentration increased relative to VP40, indicating a reduction in VLP secretion (Figure 1, D–G). GP incorporation into VLPs was comparable at 1:2 to 1:5 ratios of VP40/GP, with slightly elevated levels observed at a 1:3 ratio. Despite slightly decreased VP40 levels in VLPs, we chose a 1:3 ratio of VP40/GP for in vivo studies, considering that GP is the primary immunogen.

**Figure 2:**
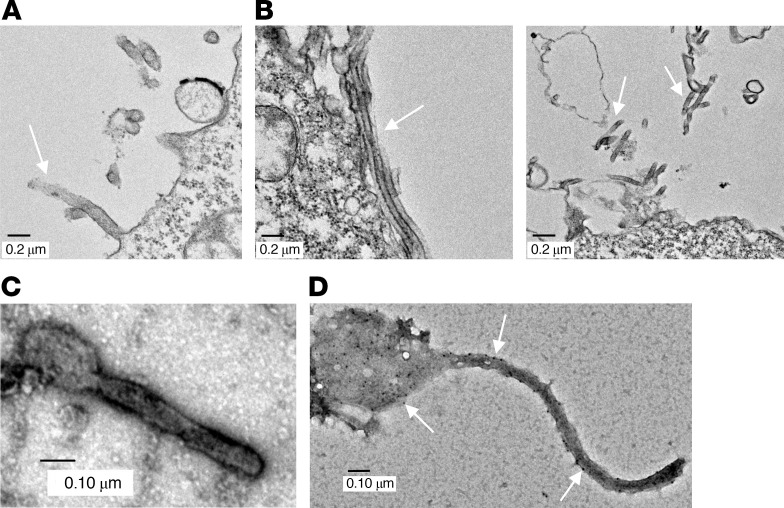
Electron microscopy of MARV VLPs. (**A**) HEK293T cells transfected with GP mRNA (control). The arrow indicates a membrane protrusion. Scale bar: 0.2 μm. (**B**) VLPs in HEK293T cells transfected with GP and VP40 mRNA. The arrows indicate VLP structures. Scale bars: 0.2 μm. (**C**) Negative staining of VLPs. Scale bar: 0.10 μm (**D**) Immunogold and negative staining of VLPs. The arrows indicate gold nanoparticle deposition. Scale bar: 0.10 μm.

VLPs were visualized using transmission electron microscopy (TEM) on ultrathin sections of HEK293T cells coexpressing GP and VP40 mRNA or GP mRNA alone (control). VLPs were observed as filamentous structures, with an approximate diameter of 60 nm and were absent on GP-only mRNA–transfected cells (Figure 2, A and B). Moreover, negatively stained, sucrose cushion–concentrated VLPs formed a club-shaped structure resembling the actual structure of MARV (Figure 2C). Immunogold staining with MR235 anti-GP antibody resulted in gold nanoparticle deposition on VLPs, confirming the incorporation of GP proteins (Figure 2D).

### MARV VP40 increases the levels of membrane-bound GP in mRNA-transfected cells.

**Figure 3:**
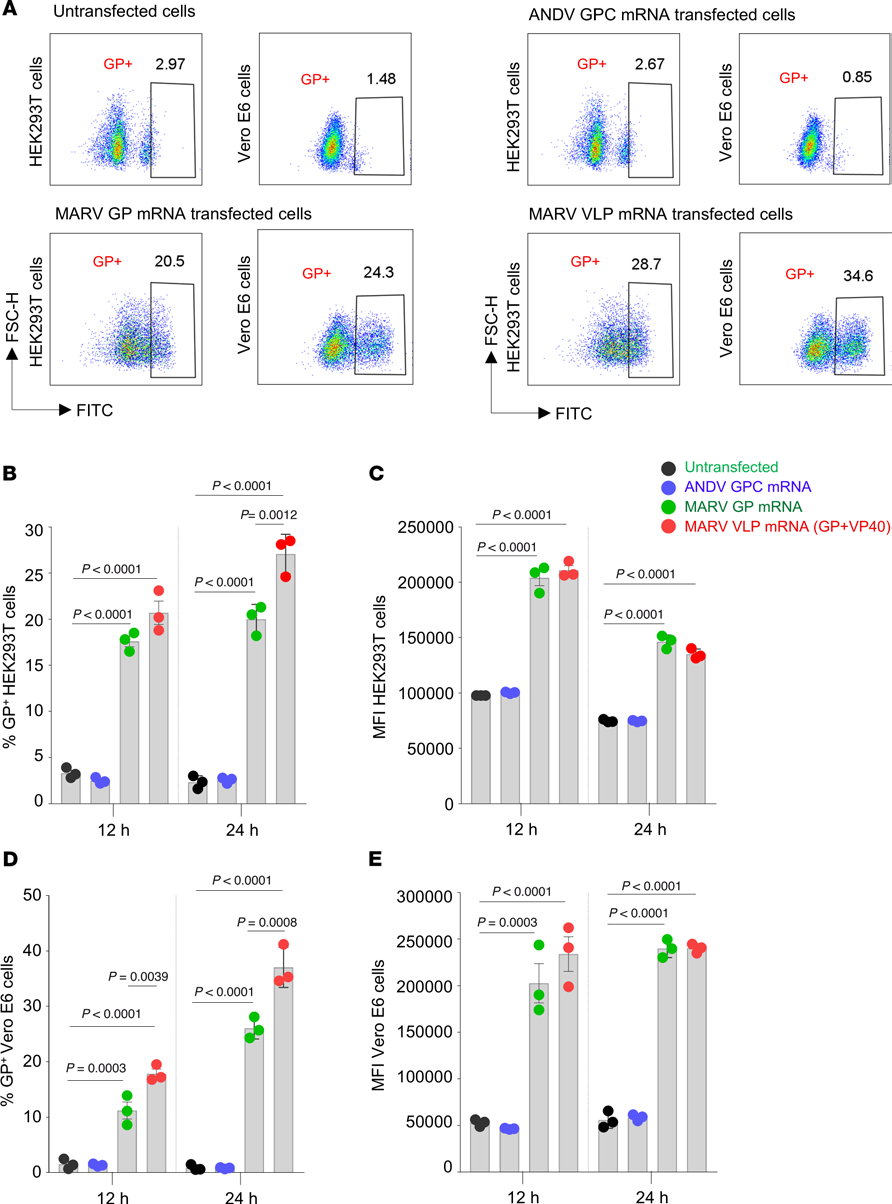
Comparison of surface staining of membrane-bound GP protein levels in mRNA-transfected cells. (**A**) Representative flow cytometry plots for a 24-hour incubation. (**B**) Percentages of GP^+^ HEK293T cells. (**C**) MFI of GP^+^ HEK293T cells. (**D**) Percentages of GP^+^ Vero E6 cells. (**E**) MFI of GP^+^ Vero E6 cells. Data are presented as the mean ± SEM. Statistical significance was calculated by 1-way ANOVA analysis followed by Tukey’s multiple-comparison test.

It was shown that membrane-bound forms of the HA of influenza virus (39) and receptor-binding domain (RBD) of SARS-CoV-2 (40) induce stronger immune responses than their soluble counterparts. MARV VP40 is a key component in virus assembly and budding, which is enriched at the plasma membrane and plays an indispensable role in recruiting and assembling GP and NP to form mature virions (41–43). Therefore, we sought to determine whether the expression of GP together with VP40 increases membrane-bound GP. HEK293T or Vero E6 cells were transfected with 300 ng GP mRNA or were cotransfected with 300 ng GP mRNA and 100 ng VP40 mRNA. Twelve and 24 hours after transfection, the cells were surface stained with a GP antibody cocktail of MR191, MR228, and MR235 (38), and flow cytometric analysis was performed (Figure 3A and Supplemental Figure 1; supplemental material available online with this article; https://doi.org/10.1172/JCI194586DS1). Cells transfected with 300 ng ANDV glycoprotein precursor (GPC) mRNA (34) were used as a negative control. Membrane-bound GP was detected in GP and VLP mRNA–transfected cells 12 and 24 hours after transfection. Although VP40 did not affect the MFI levels of GP expression, it did significantly increase the percentages of GP^+^ cells compared with cells lacking VP40 (Figure 3, B–E). Altogether, the flow cytometric data indicate that GP incorporation into cells was improved when delivered in the form of VLPs, which may be a beneficial factor in enhancing GP-mediated immune responses.

### GP and VLP mRNA vaccines induce a potent GP-binding IgG antibody response in guinea pigs, but their levels for GP mRNA are marginally higher.

**Figure 4:**
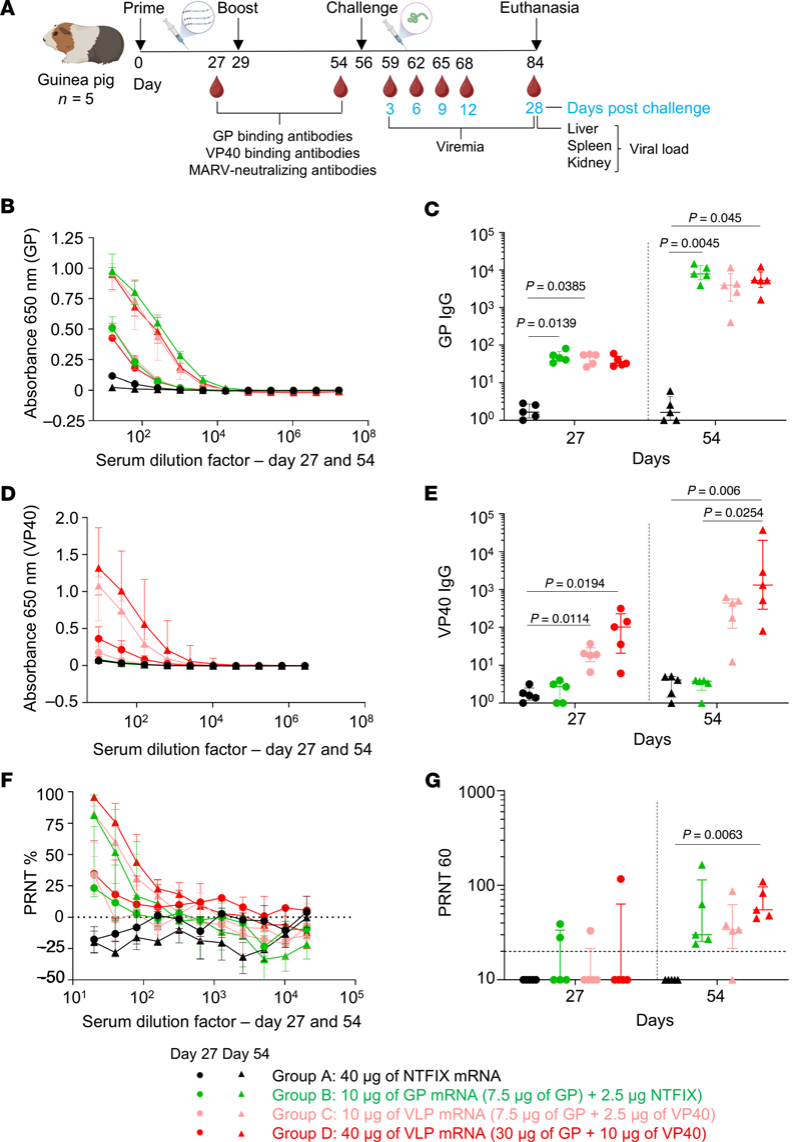
Assessment of MARV GP and VLP mRNA vaccines in guinea pig study 1: immunogenicity. (**A**) Study design: guinea pigs (*n = 5*) were vaccinated with GP mRNA (green) or VLP mRNA (red) via the intramuscular route on days 0 and 29. Serum samples were collected on days 27 and 54. On day 56, guinea pigs were challenged with guinea pig–adapted MARV, and serum samples were collected on the indicated days for viremia analysis. The study was terminated on post-challenge day 28. Schematic was created with BioRender. (**B**) MARV GP-specific ELISA absorbance values. (**C**) GP IgG antibody titers. (**D**) MARV VP40-specific binding ELISA absorbance values. (**E**) VP40 IgG antibody titers. (**F**) MARV-neutralizing antibody responses expressed as a percentage of the plaque count reduction. (**G**) PRNT_60_ titer. Data are presented as the median and IQR (**B**–**G**). Statistical significance was calculated by Kruskal-Wallis analysis followed by Dunn’s multiple-comparison test (**C**, **E**, and **G**).

We assessed the immunogenicity and protective efficacy of GP and VLP mRNA vaccines in Dunkin-Hartley guinea pigs. The VLP mRNA vaccine was a coformulation of the predetermined 1:3 ratio of VP40/GP mRNA in lipid nanoparticles (LNPs). The GP-only mRNA LNP vaccine was coformulated with nontranslated factor IX (NTFIX) mRNA to match the mRNA content delivered to animals. In study 1, nine-week-old guinea pigs were vaccinated on days 0 and 28 with 10 μg GP mRNA (GP 7.5 μg plus NTFIX 2.5 μg), 10 μg VLP mRNA (GP 7.5 μg plus VP40 2.5 μg), and 40 μg VLP mRNA (GP 30 μg plus VP40 10 μg) (Figure 4A).

Four weeks after priming and boosting, serum samples were collected to evaluate humoral responses by ELISA and a plaque reduction neutralization test (PRNT). Priming induced a comparable, low GP-binding IgG response in all vaccinated groups (Figure 4, B and C) which increased strongly following boosting. Interestingly, both 10 μg and 40 μg of the VLP vaccine elicited marginally lower GP-binding IgG responses than did the GP-alone vaccine. Median GP-specific IgG titers were 7,887 (IQR: 5,562–13,153) for the 10 μg GP–vaccinated group; 3,933 (IQR: 1,487–8,007) for the 10 μg VLP–vaccinated group; and 5,294 (IQR: 3,428–8,941) for the 40 μg VLP–vaccinated group. MARV VP40-binding IgG was detectable only in the VLP mRNA–vaccinated groups (Figure 4, D and E). Therefore, the ELISA data suggest that the GP vaccine elicited slightly elevated GP-binding IgG titers compared with the VLP vaccine.

After priming, not all guinea pigs produced neutralizing antibodies. After boosting, we detected titers in all guinea pigs, albeit at low levels, except for in 1 animal in the 10 μg VLP–vaccinated group (Figure 4, F and G). The PRNT_60_ median titers were 30 (IQR: 25–114) for the 10 μg GP; 34 (IQR: 21–62) for the 10 μg VLP; and 55 (IQR: 46–96) for the 40 μg VLP mRNA–vaccinated groups. However, the difference reached statistical significance only for the 40 μg VLP–vaccinated group compared with the NTFIX group. Thus, despite the reduced GP IgG response in the 40 μg VLP group, this group had slightly elevated levels of neutralizing antibodies compared with the GP mRNA–vaccinated group. It should be noted that the significant neutralization observed in the 40 μg vaccination group reflects its 5-fold higher GP mRNA dose rather than a vaccine-specific effect. A dose of 10 μg GP or VLP mRNA induced equivalent neutralizing antibody responses, suggesting no benefit from the VLP formulation. Altogether, the antibody data suggest that GP and VLP mRNA vaccines primarily induced GP-binding IgG responses with limited neutralizing antibodies. Importantly, the GP vaccine induced slightly higher GP-binding IgG titers than did the VLP vaccine.

### Higher doses of both mRNA vaccines equally protect guinea pigs from death and disease caused by MARV.

**Figure 5:**
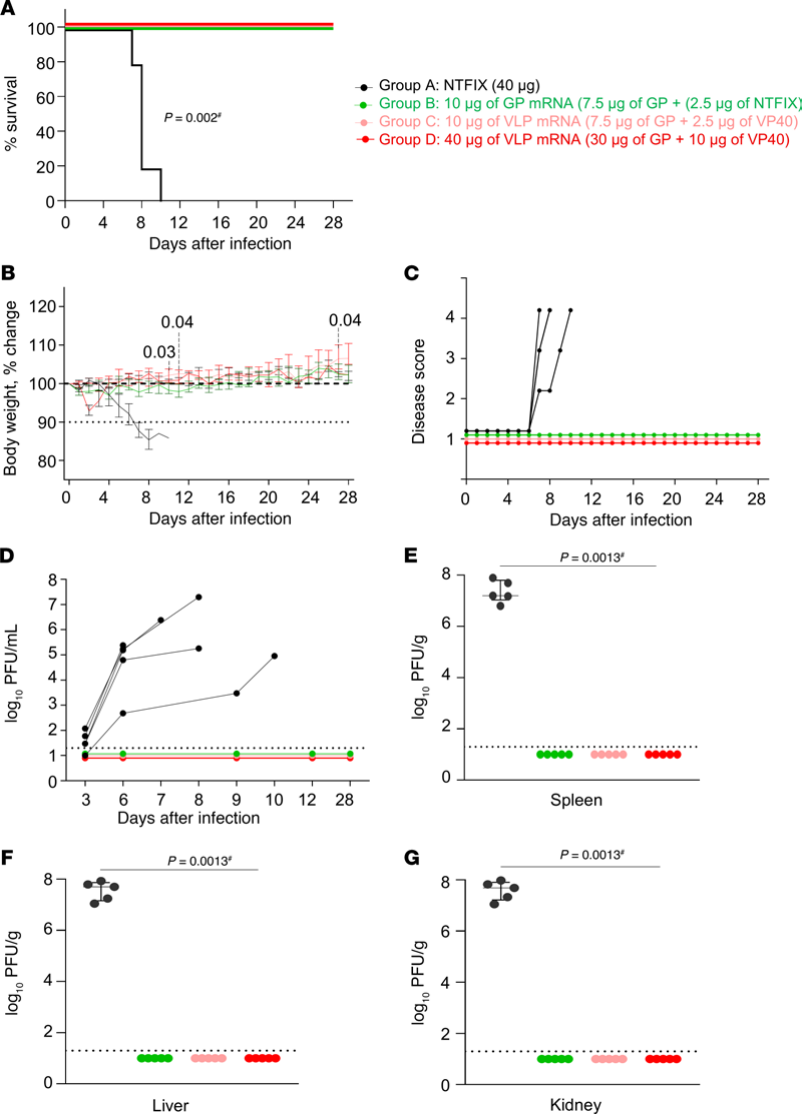
Assessment of MARV GP and VLP mRNA vaccines in guinea pig study 1: protective efficacy. (**A**) Survival curve. (**B**) Body weight percentage change. (**C**) Disease score. (**D**) Viremia. (**E**–**G**) Viral load in the spleen, liver, and kidneys, respectively. Data are presented as individual values (**A**, **C**, and **D**) and the median and IQR (**B**, **E**–**G**). The log-rank (Mantel-Cox) test was used to analyze survival data. Statistical significance was calculated by 2-way ANOVA followed by Dunnett’s multiple-comparison test (**B**) and the Kruskal-Wallis test followed by Dunn’s multiple-comparison test (**E**–**G**). ^#^Same *P* value for all vaccinated groups versus NTFIX (control).

Four weeks after boosting, the guinea pigs were challenged i.p. with 1,000 PFU of the guinea pig–adapted MARV variant Angola. Throughout the infection period, body weight, temperature, and disease scores were recorded daily until the day-28 study endpoint. Weight loss, appearance, posture, and neurological symptoms were taken into account in determining the disease score. A disease score of 1 indicated that the guinea pig was healthy and showed no signs of illness. Animals that had a score of 4 based on clinical signs of disease or greater than 20% weight loss met the euthanasia criteria. Serum was collected every 3 days through day 12, and upon euthanasia on day 28, blood, livers, spleens, and kidneys were collected for viral load quantitation by plaque assay. By day 4 after infection, most NTFIX-vaccinated guinea pigs started losing weight, which progressed to 15% below baseline, developed hyperthermia, and had high disease scores; the guinea pigs succumbed or were found moribund and euthanized on days 7–10 (Figure 5, A–C, and Supplemental Figure 2A). Serum samples from the control group tested positive for infectious virus as early as 3 days after infection (Figure 5, D–G). Terminal blood samples from the control group showed high viral titers. The control group’s splenic, liver, and kidney tissue samples also had high viral loads. In contrast, all vaccinated groups showed 100% protection against death and disease and maintained a disease score of 1, normal body temperature, and weight. The 10 μg VLP group had a marginal but significant increase in body weight compared with the 10 μg GP group on days 10, 11, and 28 (*P* < 0.03, *P* < 0.04, and *P* < 0.04, respectively). All vaccinated groups had no detectable infectious virus in serum or tissue. The data indicate that both GP and VLP mRNA vaccines conferred robust protection against the lethal challenge with MARV despite low or no neutralizing antibody levels.

### mRNA vaccines elicit potent antibody responses even at the lowest dose, but their levels are slightly elevated in the GP mRNA vaccine group.

**Figure 6:**
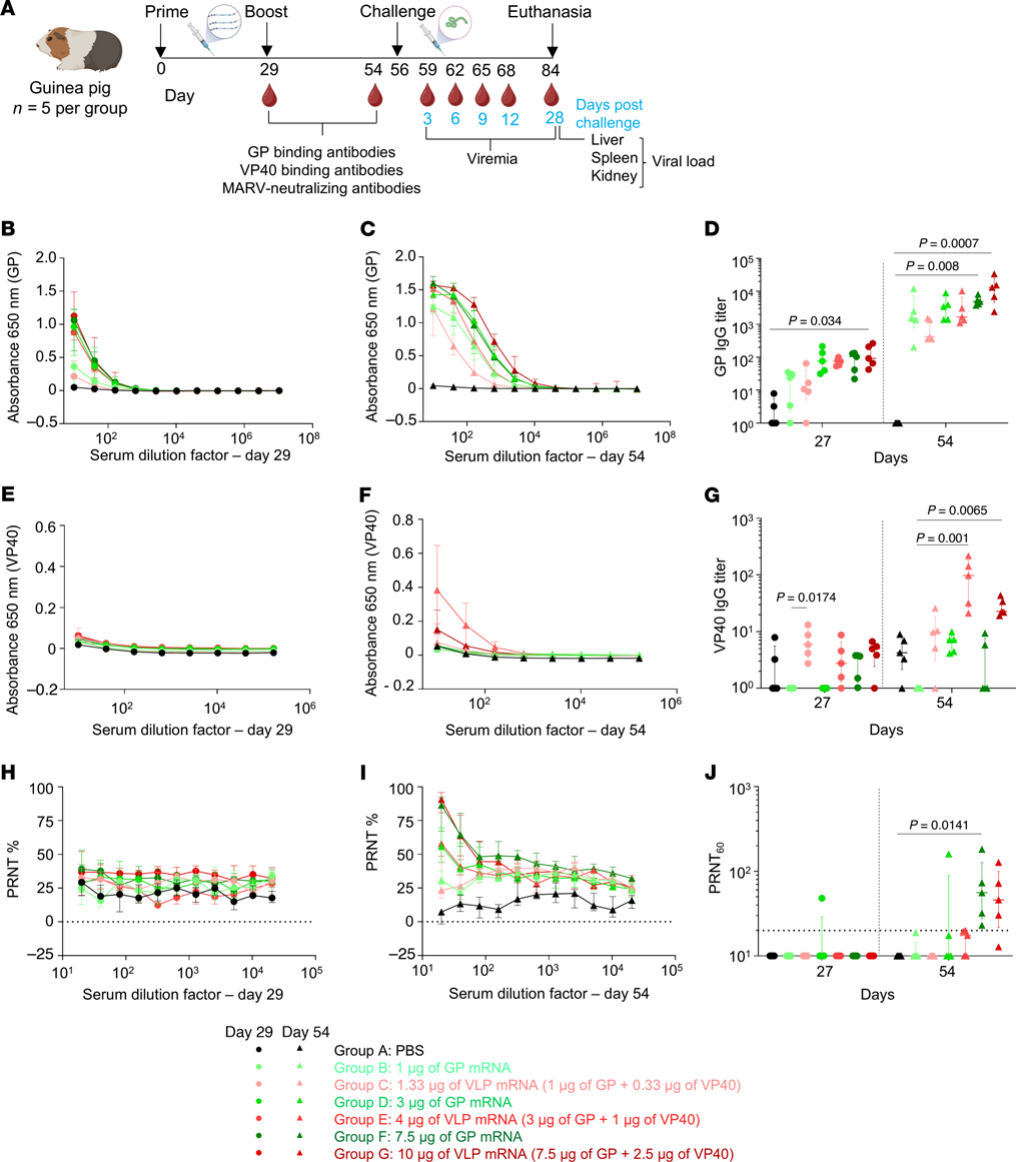
Assessment of MARV GP and VLP mRNA vaccines in guinea pig study 2: immunogenicity. (**A**) Study design: guinea pigs (*n = 5*) were vaccinated with GP mRNA (green) or VLP mRNA (red). Study 2 was conducted as described in study 1. Schematic was created with BioRender. (**B** and **C**) MARV GP–specific ELISA absorbance values. (**D**) GP IgG antibody titers. (**E** and **F**) MARV VP40–specific binding ELISA absorbance values. (**G**) VP40 IgG antibody titers. (**H** and **I**) MARV-neutralizing antibody responses expressed as a percentage of the plaque count reduction. (**J**) PRNT_60_ titer. Data are represented as the median and IQR (**B–J**). Statistical significance was calculated by Kruskal-Wallis analysis followed by Dunn’s multiple-comparison test (**D, G**, and **J**).

To better compare the 2 vaccine platforms, we tested the GP and VLP mRNA vaccines at reduced doses. In study 2, the guinea pigs were vaccinated with GP mRNA at 1 μg and 3 μg doses and VLP mRNA at doses of 1.33 μg (1 μg GP plus 0.33 μg VP40) and 4 μg (3 μg GP plus 1 μg VP40) doses (Figure 6A). In addition, the protective doses of GP at 7.5 μg and VLP at 10 μg (7.5 μg GP plus 2.5 μg VP40) mRNA from study 1 were included again as positive controls. The control group received only PBS.

GP-binding IgG antibodies were detectable after priming, even in the 1 μg GP and 1.33 μg VLP mRNA vaccinated groups, and their levels increased after boosting. Post-booster serum samples showed that 1 μg and 3 μg GP mRNA vaccines induced more than 3- and 2-fold GP IgG levels, respectively, compared with their counterparts in the VLP mRNA group. The median IgG titers were 1,518 (IQR: 783–7,217) for the 1 μg GP mRNA–vaccinated group; 418 (IQR: 356–1,452) for the 1.33 μg VLP mRNA–vaccinated group; 3,477 (IQR: 1,400–6,444) for the 3 μg GP mRNA–vaccinated group; and 1,664 (IQR: 1,225–6,666) for the 4 μg VLP mRNA–vaccinated group (Figure 6, B–D). However, 10 μg VLP mRNA induced nearly 3-fold higher IgG levels than did 7.5 μg GP mRNA. The median IgG titers were 4,922 (IQR: 3,966–6,801) for the 7.5 μg GP group and 13,207 for the 10 μg VLP group (IQR: 4,685–13,207). Except for this outlier, every GP dose in both studies elicited marginally higher GP IgG titers than did the VLP counterparts. Although the GP IgG titers induced by the identical mRNA doses in both studies differed, statistical analysis revealed no significant difference (Supplemental Figure 3, A and B). As expected, the GP-only vaccine groups had no detectable VP40 IgG antibodies (Figure 6, E–G). After boosting, VP40-binding IgG antibodies were detectable in the 4 μg and 10 μg VLP mRNA–vaccinated groups but not in the 1.33 μg VLP mRNA–vaccinated group. Of note, VP40 IgG levels were slightly higher in the 4 μg VLP–vaccinated group than in the 10 μg group. Only the highest doses of GP and VLP mRNA induced neutralizing antibodies after the booster (Figure 6, H–J), except for 1 guinea pig in the 3 μg GP group. These data indicate that even the lowest mRNA doses elicited a robust GP-binding IgG response, but levels were slightly higher in the GP mRNA–vaccinated groups than in the VLP mRNA–vaccinated groups.

### GP mRNA, but not VLP mRNA, confers complete protection at low doses.

**Figure 7:**
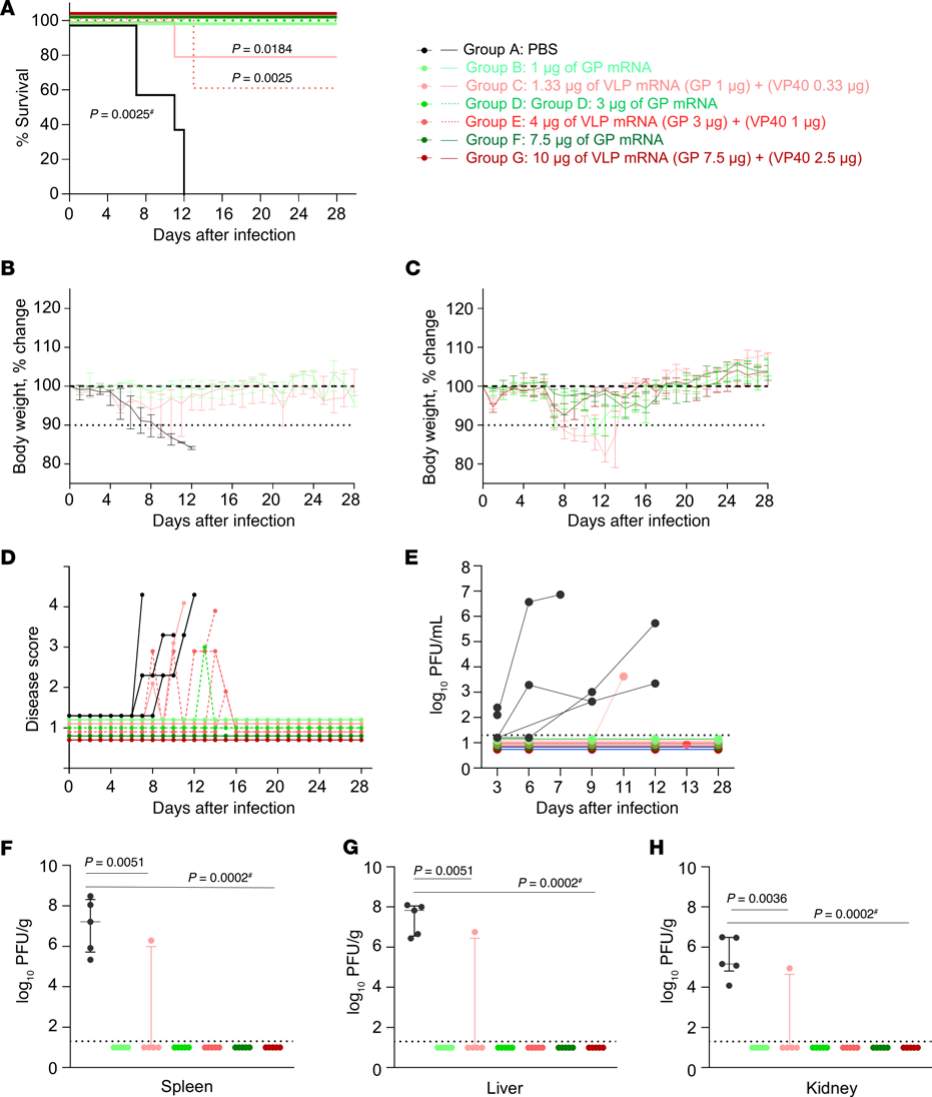
Assessment of MARV GP and VLP mRNA vaccines in guinea pig study 2: protective efficacy. (**A**) Guinea pig survival curve. (**B** and **C**) Percentage of body weight change. (**D**) Disease score. (**E**) Viremia. (**F**–**H**) Viral load in the spleen, liver, and kidneys, respectively. Data are represented as individual values (**A**–**E**) and the median and IQR (**F**–**H**). The log-rank (Mantel-Cox) test was used to analyze the survival data. Statistical significance was calculated by 2-way ANOVA followed by Dunnett’s multiple-comparison test (**B**) and the Kruskal-Wallis test followed by Dunn’s multiple-comparison test (**F**–**H**). ^#^Same *P* value for all vaccinated groups versus the PBS control; otherwise, the specific *P* values are indicated.

Four weeks after the boost vaccination, guinea pigs were challenged with the guinea pig–adapted MARV as described in study 1. The PBS control group developed disease signs and lost up to 15% of their body weight; they succumbed to infection on days 7–12 (Figure 7, A–D). The control group also experienced hyperthermia followed by hypothermia (Supplemental Figure 2B). Consistent with clinical signs and high disease scores, serum and tissue samples (spleen, liver, kidney) showed a high viral load (Figure 7, E–H). On the other hand, all guinea pigs that received GP mRNA vaccine doses (1, 3, 7.5 μg) and those that received the highest VLP mRNA vaccine dose (10 μg) groups survived, and all guinea pigs but 1 in the 3 μg GP group showed no outward signs of illness. The animals maintained normal body temperature (Supplemental Figure 2C), had no or a low disease score, gained weight throughout the study, and had no detectable viremia or viral load in organs. In contrast, the 1 μg and 4 μg VLP mRNA–vaccinated groups had 80% and 60% protection, respectively. The guinea pigs that died lost body weight and had high disease scores. In addition, all surviving guinea pigs in the 4 μg VLP group showed signs of disease with a score of 3 before recovering. Viral load analysis revealed that the nonsurvivor in the 1 μg group had high levels of infectious virus in serum and tissue samples, whereas the 2 nonsurvivors in the 4 μg VLP group had no detectable virus.

**Figure 8:**
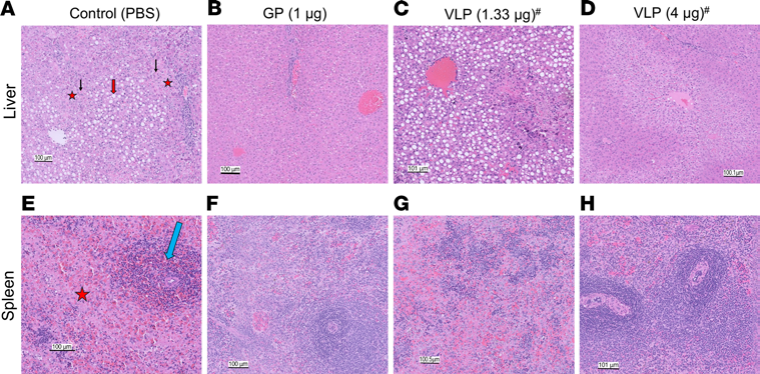
Histopathology of liver and spleen tissues from control and vaccinated groups from the dose-down study. Liver and spleen tissues collected from the guinea pigs were stained with H&E. ^#^For the VLP group (1.33 μg and 4 μg), the images represent the guinea pigs that succumbed to infection. Scale bars: 100 μm. (**A**) Control liver section shows the following characteristics: apoptosis/necrosis (red asterisks), hepatocellular vacuolation (red arrow), and councilman-like bodies (black arrows). (**B–D**) Liver sections from the indicated vaccine groups. (**E**) The control spleen section shows apoptosis/necrosis in the red pulp (red star) and lymphocyte depletion in the white pulp (blue arrow). (**F**–**H**) Spleen sections from the indicated vaccine groups.

In the PBS control group, liver tissue sections showed characteristic histopathologic changes, including hepatocellular vacuolation, sinusoidal leukocytosis, apoptosis/necrosis, focal cytoplasmic inclusions, and occasional councilman-like bodies (Figure 8A). In contrast, samples from the vaccinated groups displayed normal liver histology except for the sample from the nonsurvivor in the 1 μg VLP group, which had histopathologic changes similar to those seen in the PBS group (Figure 8, B and C). However, samples from nonsurvivors in the VLP 4 μg group exhibited normal histology with a small amount of glycogen deposition in the liver (Figure 8D). Similarly, spleen sections from the control group showed lymphocyte depletion in the white pulp, necrosis, and inflammation in the red pulp with diffuse hemorrhaging and tangible-body macrophages (Figure 8E). Spleen sections from all vaccinated groups showed normal histology except in the 1 nonsurvivor in the 1 μg VLP (Figure 8, F–H).

The guinea pig study 1 demonstrated that 7.5 μg GP and 10 μg VLP mRNA vaccines completely protected against MARV infection. In line with this, the complete protection conferred by high vaccine doses in study 2 highlights the reproducibility and robustness of mRNA vaccine–induced protective responses against MARV. These data also show that the lowest GP mRNA vaccine dose tested — 1 μg — provided 100% protection against death and disease caused by a lethal dose of MARV. In contrast, the same dose of GP mRNA vaccine combined with 0.33 μg VP40 mRNA conferred 80% protection. Histology and viral load data demonstrated that the guinea pig given 1 μg VLP succumbed due to the infection. Guinea pigs in the 3 μg GP mRNA group showed 100% protection, but their VLP counterparts showed 60% protection; the lack of detectable viral load and histopathological data indicated that the reduced protection in the 4 μg VLP group was unrelated to viral infection or it could be an unknown VLP vaccine–induced effect.

### The VLP mRNA vaccine induces a subtle GP-specific CD8^+^ T cell predominance, whereas the GP mRNA induces a modestly skewed CD4^+^ T cell response.

**Figure 9:**
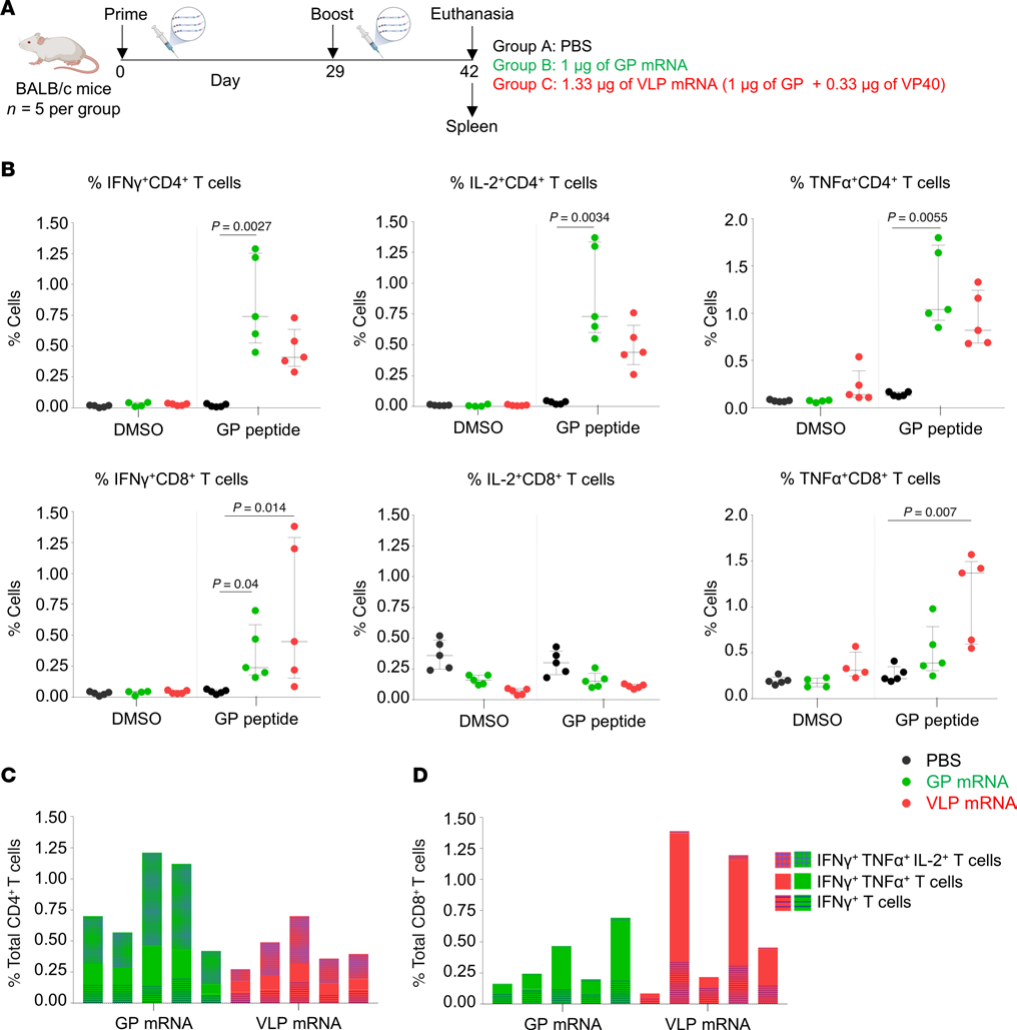
MARV GP–specific T cell responses to GP and VLP mRNA vaccines. (**A**) Study design: 6- to 7-week-old female BALB/c mice (*n = 5*) were vaccinated with GP mRNA (green) or VLP mRNA (red) via the intramuscular route on days 0 and 29. Control mice received PBS (black). Spleens were collected and processed to isolate splenocytes. Splenocytes were stimulated with DMSO or GP-peptide pool to measure GP-specific CD4^+^ and CD8^+^ T cells by flow cytometry. Schematic was created with BioRender. (**B**) Percentages of the indicated cell populations. (**C**) Percentages of total CD4^+^ T cells producing the indicated cytokines. Each bar indicates the value for the individual percentage of CD4^+^ T cells for each mouse. (**D**) Percentages of total CD8^+^ T cells producing the indicated cytokines. Each bar indicates the value for the individual percentage of CD8^+^ T cells for each mouse. Data are presented as the median and IQR (**B**–**G**) and values for individual animals (**C** and **D**). Statistical significance was calculated by Kruskal-Wallis analysis followed by Dunn’s multiple-comparison test (**B**–**G**).

The low-dose VLP mRNA vaccine conferred incomplete protection compared with the GP-alone mRNA vaccine, which could be attributed to the lower GP-binding IgG antibody titers. We investigated whether these vaccines induce differential CD8^+^ and CD4^+^ T cell responses. To address this, 6- to 7-week-old female BALB/c mice were vaccinated on days 0 and 29 with 1 μg GP mRNA, 1.33 μg VLP mRNA (GP 1 μg plus VP40 0.33 μg), or PBS (control) (Figure 9A). Two weeks after boosting, MARV GP-specific CD4^+^ and CD8^+^ T cell responses were measured in splenocytes by flow cytometry (Supplemental Figure 4). The cells were stimulated with a pool of 15 amino acid long peptides, which cover the full-length GP of the MARV Angola strain with an 11 amino acid overlap, and then surface stained for CD4 and CD8 and intracellularly stained for IFN-γ, IL-2, and TNF-α. The control cells were mock stimulated with DMSO (Figure 9B). The GP mRNA vaccinated group had significantly more CD4^+^ T cells positive for IFN-γ, IL-2, or TNF-α when compared with the control PBS group. Although the VLP-vaccinated group showed GP-specific CD4^+^ T cell responses, the difference to the PBS-vaccinated control group did not reach statistical significance. Compared with PBS controls, both GP and VLP mRNA groups showed a significant increase in IFN-γ^+^CD8^+^ T cells, but only VLP mRNA induced a significant increase in TNF-α^+^CD8^+^ T cells (*P* = 0.04 for GP mRNA; *P* = 0.014 for VLP mRNA), but only VLP mRNA induced a significant increase in TNF-α^+^CD8^+^ T cells (*P* = 0.007). The GP mRNA vaccine induced more triple-positive CD4^+^ T cells coproducing IFN-γ, IL-2, and TNF-α, whereas the VLP mRNA vaccine induced more double-positive CD8^+^ T cells coproducing IFN-γ and TNF-α (Figure 9, C and D). However, none of the CD4^+^ or CD8^+^ T cell populations differed significantly when we compared GP and VLP groups directly. Altogether, our data suggest that both mRNA vaccines induced GP-specific T cell responses with a modest skewing toward CD4^+^ T cells in the GP mRNA group and CD8^+^ T cells in the VLP mRNA group.

### The VLP mRNA vaccine, but not the GP-only vaccine, changes the guinea pig whole-blood transcriptome.

To understand transcriptional changes in the 4 μg VLP guinea pig group, which had reduced survival despite the protection against viral replication, we performed bulk RNA-Seq of guinea pig whole blood on post-vaccination day 54 and post-challenge day 3. We included the following groups: PBS control, 3 μg and 7.5 μg GP mRNA, and 4 μg and 10 μg VLP mRNA.

**Figure 10:**
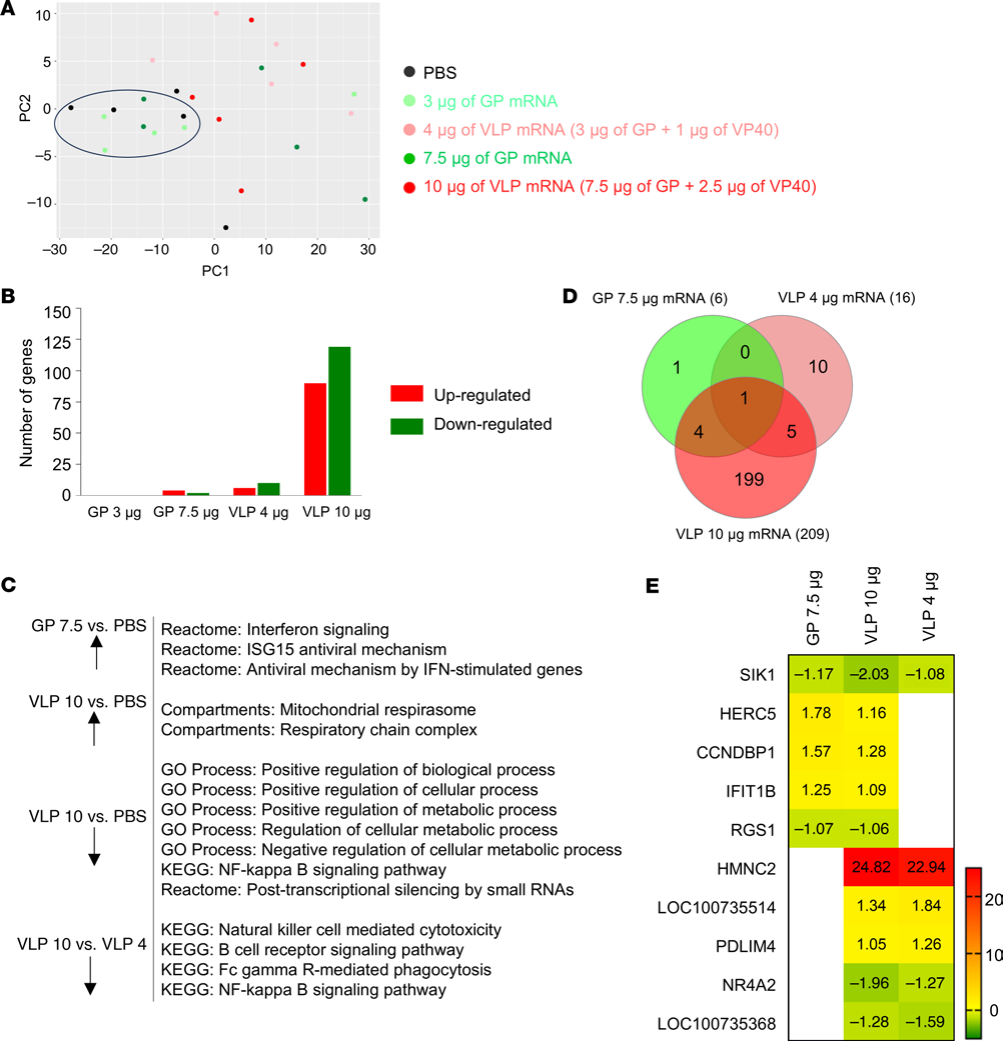
GP mRNA vaccine induces minimal changes in the guinea pig whole blood transcriptome compared with the VLP mRNA vaccine. (**A**) PCA of genes expressed in the control and vaccinated groups**.** (**B**) Total number of up- and downregulated genes in the whole blood of vaccinated groups versus PBS. The graph shows genes with a log_2_(fold change) of 1 or greater and an adjusted *P* value of 0.05 or less. (**C**) Functional enrichment analysis of significantly differentially expressed genes. Arrows pointing up or down indicate up- and downregulated genes, respectively. (**D**) Venn diagram of overlapping genes between GP and VLP mRNA–vaccinated groups versus PBS. (**E**) List of overlapping genes between the GP and VLP mRNA–vaccinated groups versus PBS with log_2_(fold change) values.

Principal component analysis (PCA) of day 54 post-vaccination samples showed that the PBS and 3 μg GP–treated groups clustered together, unlike the 4 μg and 10 μg VLP–vaccinated groups (Figure 10A). In addition, 2 samples from the 7.5 μg GP group were also associated with the PBS/3 μg GP cluster. This suggests that GP mRNA induced minimal changes in the transcriptome, whereas VLP mRNA induced marked changes. Genes with a log_2_(fold change) of greater than 1 and a *P* value of 0.05 or less showed that the GP-vaccinated group had no differential expression (3 μg GP) or very low differential expression (7 μg GP) compared with the PBS group (Figure 10B). In contrast, the 10 μg VLP and 4 μg VLP groups had 209 and 16 significantly differentially expressed genes, respectively (Supplemental Data 1).

Functional enrichment analysis showed upregulation of reactome terms for IFN signaling in the 7.5 μg GP group (Figure 10C). However, the 10 μg VLP group had downregulated genes corresponding to Gene Ontology (GO) terms for biological and metabolic processes, including the NF-κB signaling pathway and posttranscriptional silencing by small RNAs; the majority of these downregulated genes interact with each other and form a highly connected protein-protein interaction network (Supplemental Figure 5). In addition, genes involved in the mitochondrial respiratory chain complex were upregulated (Supplemental Data 1). Surprisingly, the comparison of 10 μg VLP versus 4 μg VLP showed downregulation of NK cell–mediated cytotoxicity, B cell receptor signaling, and the NF-κB pathway. A closer look at the gene list revealed that the 10 μg VLP group had genes overlapping with those of the 4 μg VLP and 7.5 μg GP groups (Figure 10, D and E). Among the overlapping genes, we found that 7.5 μg GP and 10 μg VLP mRNA commonly upregulated immune-related genes (Supplemental Table 1), HECT and RLD domain–containing E3 ubiquitin-protein ligase (*HERC5*), cyclin D1 binding protein 1 (*CCNDBP1*), and IFN-induced protein with tetratricopeptide repeats 1B (*IFIT1B*) (44–46), which were not induced in the 4 μg VLP group. Interestingly, both VLP doses showed upregulated S100 family protein linked to poor prognosis in Ebola virus disease (47) and COVID-19 (48). Overall, the data suggest that GP mRNA induced a minimal effect on the blood transcriptome, whereas VLP mRNA induced more changes. In addition, the high-dose VLP vaccine downregulated the expression of genes involved in the immune response.

**Figure 11:**
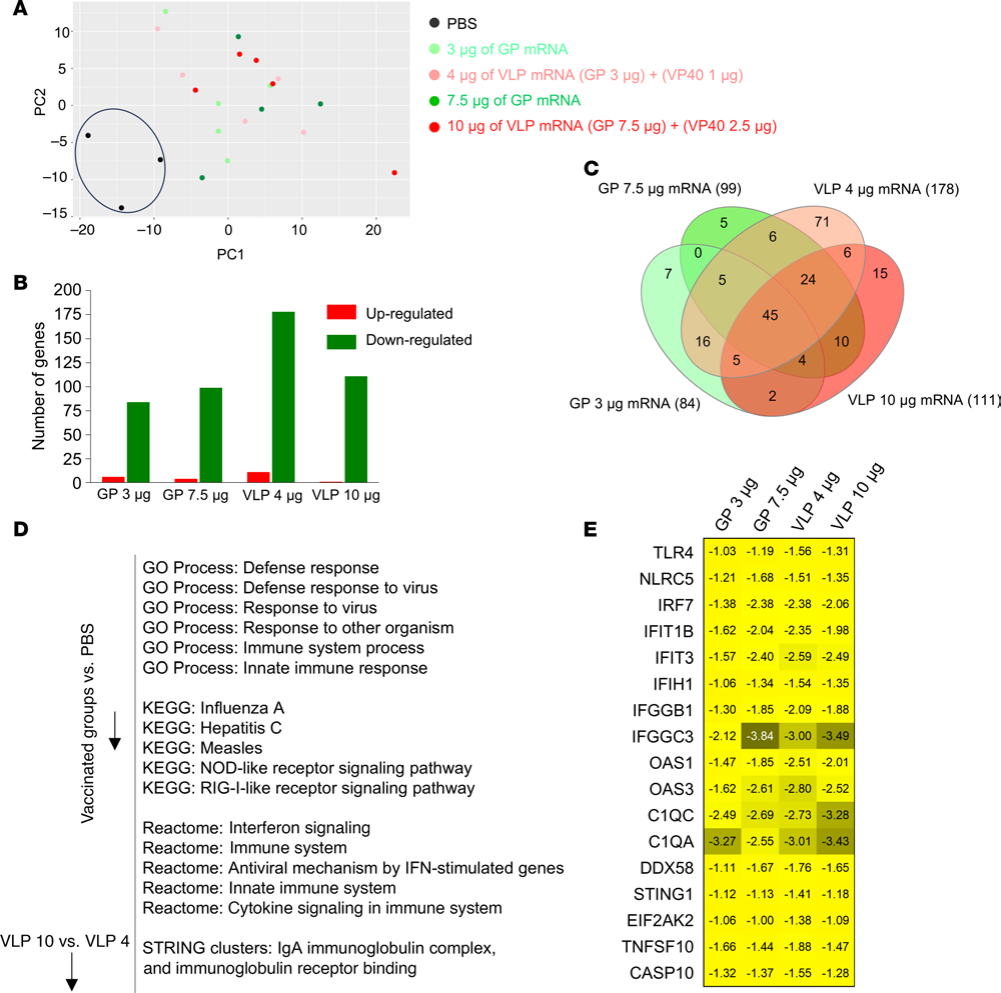
Vaccinated guinea pig groups show reduced expression of antiviral response genes compared with the unvaccinated group. (**A**) PCA of genes expressed in control and vaccinated groups. The 2 outlier samples from the control group were removed for better visualization. (**B**) Total numbers of up- and downregulated genes in the whole blood of vaccinated groups versus PBS treatment. The graph shows genes with a log_2_(fold change) of 1 or greater and an adjusted *P* value of 0.05 or less. (**C**) Venn diagram showing the intersection of overlapping downregulated genes among all vaccinated groups versus PBS. The numbers in parentheses indicate the total number of genes in the indicated group. The numbers in parentheses indicate the total number of genes in the indicated group. (**D**) Functional enrichment analysis of overlapping downregulated genes. (**E**) Genes related to the immune response and apoptosis with log_2_(fold change) values.

Next, we characterized the differential gene expression of GP and VLP mRNA–vaccinated groups in day 3 post-challenge blood samples. PCA indicated that the results for the PBS group were separate from those for all vaccinated groups (Figure 11A). Vaccinated groups showed downregulation of 84, 99, 178, and 111 genes in the 3 μg GP, 7.5 μg GP, 4 μg VLP, and 10 μg VLP–vaccinated groups, respectively, as compared with the PBS control group (Figure 11B and Supplemental Data 2), with 45 overlapping genes (Figure 11C). These 45 genes were enriched for the viral immune response and cytokine signaling terms and pathways (Figure 11D). These included pathways and signaling associated with TLR, nod-like receptor (NLR), IFN, complement,and apoptosis (Figure 11E). This demonstrates that the vaccinated groups had reduced levels of immune response pathways due to vaccine-mediated protective effects. Of note, the 4 μg VLP group had 71 unique downregulated genes. The analysis of these unique genes did not indicate pathways correlated with the reduced protection. However, it is tempting to speculate that the downregulation of a distinct set of genes in the 4 μg VLP group was probably associated with the reduced protection in this group. Last, a comparison of 10 μg VLP versus 4 μg VLP revealed downregulation of Ig receptor–binding genes. These data suggest that unprotected control guinea pigs had exaggerated immune responses upon MARV infection, characterized by upregulation genes involved in cytokine signaling, whereas the vaccinated guinea pigs showed reduced expression of these genes due to vaccine-mediated protective effects.

## Discussion

In this study, we developed and assessed the protective efficacy of mRNA vaccines against MARV and assessed whether the MARV VLP vaccine provides an added advantage over the GP-only vaccine. Previous studies showed that protein- or viral vector–derived MARV VLP vaccines are protective in small animals and NHPs but lacked GP-only control and dose-ranging data, making it unclear whether the VLP vaccine has an edge over the GP vaccine (13, 21). The minimal component MARV strain Musoke VLP vaccine derived from cell culture shows complete protection against challenge with MARV after 3 intramuscular injections, each consisting of a 50 μg dose in guinea pigs and a 1 mg dose in NHPs (21). The present study provides a more comprehensive understanding of the efficacy of GP and VLP vaccines against MARV. Our side-by-side comparison of the VLP and GP groups showed that the VLP vaccine failed to protect guinea pigs at low doses, whereas the GP-alone vaccine conferred 100% protection even at the lowest dose. In addition, our study provides supporting evidence as to why VLP failed to confer protection.

First, GP-specific binding IgG levels were moderately reduced in the VLP-vaccinated group. The available NHP data indicate that GP-binding IgG levels correlate with protection (11, 49). In line with this, our data also showed that mRNA vaccines conferred complete protection despite no or low detectable neutralizing antibodies, suggesting that the Fc-mediated response or alternative antiviral mechanisms mediated by antibodies could play a role in protection (13, 50, 51). Therefore, the reduced protection in the VLP-vaccinated group could be due to reduced levels of GP-binding IgG. Although we reasoned that the reduced GP-binding IgG titers were one of the contributing factors, we could not establish the minimum level of antibodies required for protection due to the modest differences in either GP- or VP40-binding titers, or neutralizing antibody titers between survivors and nonsurvivors (Supplemental Tables 2 and 3). VLP-vaccinated guinea pigs indeed had a 2- to 3-fold reduction of median GP-binding IgG titers at low doses compared with the GP group; however, within the VLP group, there was no difference in antibody titers between survivors and nonsurvivors. In the same way, their VP40-specific IgG levels did not correlate with survival, and the majority of them did not elicit virus-neutralizing antibodies.

The role of T cell immunity in MARV GP-induced protective responses is not completely understood. However, irrespective of a vaccine platform, most GP vaccines have been shown to induce predominantly CD4^+^ T cell responses and lower CD8^+^ T cell responses. ChAdOx1-vectored GP in mice (16), VSV-vectored (52) and DNA encoding GP (53) in NHPs, cAd3-vectored GP (17), and DNA encoding GP (9) in phase I clinical trials have reported induction of dominant CD4^+^ T cell responses and less frequent CD8^+^ T cell responses. Conversely, recombinant Ad5-vectored GP is the only vaccine shown to induce a cellular immune response with skewing toward CD8^+^ T cells (53). Moreover, analysis of the T cell immune response from MARV infection survivors showed the development of multifunctional CD4^+^ T cell responses expressing IFN-γ and IL-2 with limited CD8^+^ T cell responses (54). In line with these data, our mouse study showed that the VLP vaccine elicited moderately elevated CD8^+^ T cell responses, whereas the GP vaccine induced CD4^+^ T cell responses. Although both vaccines induced CD4^+^ T cell responses, the GP-vaccinated group had an increase in activated CD4^+^ T cells coexpressing IFN-γ, IL-2, and TNF-α in contrast to the VLP-vaccinated group, which had slightly more CD8^+^ T cells double-positive for IFN-γ and TNF-α. Our data indicate that multifunctional CD4^+^ T cell responses could be associated with protection.

RNA-Seq of guinea pig whole blood at day 54 post-vaccination indicated that the VLP mRNA vaccine at the 10 μg dose affected gene expression associated with various metabolic and cellular processes. Specifically, genes associated with RNA-induced silencing complex complex assembly, pre-miRNA processing, RNA secondary structure unwinding, and histone H3-K4 methylation were downregulated. Incidentally, Ebola viruses VP30, VP35, and VP40 have been shown to suppress RNA silencing to antagonize RNAi-dependent antiviral immunity (55). In addition, several respiratory chain complex genes were upregulated. Intriguingly, the 10 μg dose of VLPs compared with the 4 μg dose induced downregulation of NK cell–mediated cytotoxicity, B cell receptor signaling, and NF-κB pathways. In contrast, GP vaccination induced no changes in the transcriptome in the 3 μg group, and few genes related to type I IFN (IFN-I) signaling were upregulated in the 7.5 μg GP group. Together, the transcriptome data suggest that VP40 induced substantial changes in the expression of genes in whole blood. Moreover, MARV VP40 has been shown to antagonize IFN-I signaling by inhibiting the phosphorylation of Janus kinases and STAT proteins in response to IFN-I and type II IFN, as well as IL-6 (56, 57). Hence, VP40 could exhibit some indirect effects on host immune cells, thereby affecting the immune response to GP.

It is well known that innate immune signaling is the first line of defense against viruses, including MARV (12, 58, 59). In line with this, after the challenge, unvaccinated guinea pigs showed upregulation of genes involved in pattern recognition receptors (*TLR4*, *NLRC5*), the IFN-I pathway (*IFIT1B*, *IFIH1*, *IFGGB1*, *IFGGC3*, *OAS1*, *OAS3*, *STING1*), the complement pathway (*C1QC*, *C1QA*), the apoptosis pathway (*CASP10*), and *EIF2AK2*, double-stranded, RNA-activated protein kinase. However, vaccinated animals had reduced levels of these transcripts, indicating vaccine-mediated protective effects upon MARV infection. Interestingly, in the 4 μg VLP–vaccinated group, which had 2 nonsurvivors, 178 genes were downregulated; this was nearly 2-fold higher than in the other vaccinated groups. Hence, it is impossible to rule out that VP40-mediated immunosuppressive effects could be the culprit behind the reduced survival of the 4 μg VLP mRNA group despite showing protection from viral infection. Further in vitro and in vivo studies are needed to understand how VP40 modulates host cell function.

The results from this study raise an important question: Are the VP40-mediated adverse effects restricted to MARV or do they also occur with other viruses in the filovirus family, such as Ebola virus, even though IFN-I antagonism of Ebola virus is associated with VP35 and VP24 (60) rather than VP40? One NHP study indirectly suggested that VP40-mediated effects could also be extended to Ebola virus, in which the EBOV GP-VP40 VLP (VLP 2-component) protein-derived vaccine was tested with a dose range of 25 μg to 3 mg in NHPs (61). The vaccine demonstrated only partial protection even at the highest 3 mg dose; survivors had GP with the transmembrane domain deleted titers of greater than 1,400 AU/mL and GP with the mucin-like domain deleted titers of greater than 1,900 AU/mL, whereas most nonsurvivors’ titers fell below these thresholds. In contrast, NHPs receiving a 3-component VLP (with NP also included) or an Ad-GP vaccine achieved GP titers similar to those of nonsurvivors of EBOV 2-component VLP and survived, with no symptoms. The authors concluded that the 2-component VLP vaccine requires a significantly higher GP antibody threshold for protection than does the Ad-GP or triple-component VLP vaccine. In line with this, it is possible that the MARV VLP vaccine may also require a higher GP antibody threshold for protection than does the GP-alone vaccine. Moreover, several other vaccine platforms have shown that GP-only vaccines confer complete protection to NHPs (11, 52, 62). Therefore, it is tempting to speculate that the reduced protection by the Ebola VLP vaccine could be due to VP40-mediated effects. More studies are warranted to reproduce VP40-induced adverse effects and to elucidate the mechanisms by which VP40 modulates host immunity.

While mRNA-LNP vaccine technology is now well established for single-component vaccines, its application to VLP vaccines is still in exploratory stages. Although it was successfully tested for SARS-CoV-2 (37, 63) and HIV-1 (31, 63) in preclinical studies, mRNA-derived VLP vaccine needs more studies to match gold-standard protein-derived VLP vaccines. Unlike single-component or bivalent mRNA vaccines, which require only efficient translation of 1 or 2 proteins, the VLP mRNA vaccine must accomplish a balanced coexpression of 2 or more proteins, enrichment at the plasma membrane, assembly, and VLP release. In contrast, protein-derived VLP vaccines are fully assembled and readily accessible to immune cells. Although our in vitro data demonstrated VLP assembly and release by mRNA, predicting and demonstrating in vivo VLP assembly remains challenging. This is particularly relevant because our study is the first to our knowledge to show reduced immunogenicity and protective efficacy of a VLP mRNA vaccine as compared with an envelope protein–only vaccine. Further studies are needed to compare VLP mRNA with protein-derived vaccines to ensure that the in vivo assembly bottleneck does not impair the vaccine’s efficacy.

The study’s major limitation is the multifaceted role of VL40 in MARV VLP formation, which complicates the understanding of its specific effect on GP-mediated immune modulation. VP40 oligomerizes at the inner plasma membrane to form lattice structures and recruits GP, NP, and accessory components to budding sites, facilitating MARV assembly and release (41–43). In line with this well-established data, our in vitro results showed enhanced surface GP expression, VLP assembly, and release in the presence of VP40. However, the VLP vaccine did not induce an enhanced antibody response and displayed reduced protection. It should be emphasized that GP expressed alone was partly surface expressed and secreted; in contrast, GP coexpression with VP40 enhanced surface expression and was released as VLP, altering the balance between intracellular, surface-bound, and extracellular GP. Despite the fact that we administered equal amounts of GP-encoding mRNA in guinea pigs, the resulting GP could be distributed differently across the cell, complicating the direct comparison of these vaccine formulations. Although the surface GP expression we measured could represent a transient intermediate step, and surface-expressed GP could ultimately become a part of VLP, it is noteworthy that mRNA-derived, membrane-bound immunogens lower the activation threshold for germline-targeting antibody responses (64). Therefore, the reduced protection by VLP may stem from altered GP distribution or the direct immunomodulatory effects pf VP40. Further studies are needed to dissect VP40’s specific immunomodulatory roles and to clarify how differential GP localization influences protective immunity.

In summary, we developed mRNA-based vaccines against MARV and demonstrated excellent immunogenicity and protection for the GP-only vaccine. However, the MARV VLP vaccine did not show enhanced immune responses or protection compared with the GP-only vaccine, suggesting that the presence of VP40 or the assembly of the vaccine components in VLPs has reduced the GP-mediated immune response.

## Methods

### Sex as a biological variable.

Our study exclusively examined female guinea pigs and mice. There were no indications of any significant difference between male and female guinea pigs and mice in immune responses to mRNA vaccines and between male and female guinea pigs in susceptibility to Marburg virus. The selection of female guinea pigs was also related to limited space in BSL-4 containment facilities.

### Development of the mRNA vaccines.

mRNA vaccines encoding GP and VP40 for MARV were synthesized in vitro using an optimized T7 RNA polymerase–mediated transcription reaction and formulated in LNPs as described previously (37).

### Confirmation of GP and VP40 protein expression by Western blotting.

HEK293T cells with 70%–80% confluence were individually or cotransfected with 500 ng GP and VP40 mRNA in 24-well plates using the TransIT-mRNA Transfection Kit (Mirus Bio) following the manufacturer’s guidelines. Twenty-four hours after transfection, cells were lysed in cell lysis buffer (Cell Signaling Technology, 9803) with a protease inhibitor. Proteins were quantified using the BCA Protein Assay kit (Pierce, Thermo Fisher Scientific, 23227) and equalized. Proteins were separated by NuPAGE Bis-Tris Mini Protein Gels (Invitrogen, Thermo Fisher Scientific, NP0322BOX) and transferred onto nitrocellulose membranes using the iBlot 2 Dry Blotting System (Invitrogen, Thermo Fisher Scientific, IB23001). Then, blots were incubated with a blocking buffer (LI-COR, 927-70001) and primary and secondary antibodies. Human monoclonal antibody MR235 (38) and mouse monoclonal antibody 6B1 (IBT Bioservices, 0203-016) were used to detect GP and VP40 proteins, respectively. Fluorescence-labeled secondary antibodies (LI-COR) were used and visualized using Odyssey Fc OFC-1130 (LI-COR). The fluorescence intensity of protein bands was quantified using the in-built software of Odyssey. For optimization of the GP and VP40 mRNA ratio, 0.5, 1, 2, 3, 4, and 5 μg GP mRNAs were cotransfected with 1 μg VP40 mRNA for 48 hours in HEK293T cells in 100 mm plates. VLPs were purified from culture supernatant as described below. Cell lysates and purified VLPs were analyzed using Western blotting.

### Purification of virus-like particles from culture supernatant.

As described above, 10 μg GP and VP40 mRNA were transfected in HEK293T cells in a T-75 flask. Forty-eight hours after transfection, the supernatants were clarified by centrifugation at 1,700*g* for 10 minutes at 4°C. The clarified supernatants were layered over the 25% sucrose and centrifuged in a SW32 rotor of a Beckman Coulter Optima L-90K ultracentrifuge for 2 hours at 27,000 rpm at 4°C. The pellets were dissolved in sodium chloride-tris-EDTA buffer (Fisher Bioreagents, Thermo Fisher Scientific, BP2479-1) and analyzed by Western blotting and TEM.

### Electron microscopy of MARV-like particles.

HEK293T cells were transfected with 20 μg GP or GP and VP40 mRNA in two T-75 flasks to prepare ultrathin sections for TEM imaging. The cells were fixed in a fixative buffer containing 2.5% formaldehyde, 0.1% glutaraldehyde, 0.01% picric acid (trinitrophenol), 0.03% CaCl_2_, and 0.05 M cacodylate buffer, pH 7.3–7.4, for 2–3 hours at room temperature. Then, cells were washed 3 times with 0.1 M cacodylate buffer for 10 minutes each time at room temperature, followed by incubation with 2% uranyl acetate for 20 minutes at 60°C with subsequent incubation with 50%, 75%, 95%, and 100% ethanol. Then, samples were infiltrated with propylene oxide and embedded in Poly/Bed 812 resin (Polysciences). Finally, blocks were trimmed, sectioned, and visualized under a JEM-1400 electron microscope (JEOL). For negative staining of VLPs, 10 μL purified VLPs were applied on nickel grids and incubated for 10 minutes, followed by 2% aqueous uranyl acetate treatment for 1 minute and then observed under the electron microscope. For Immunogold staining, VLPs were applied on nickel grids, followed by incubation with MR235 human monoclonal antibody and 6  nm colloidal gold goat anti-human antibody (Jackson ImmunoResearch Laboratories). Then, grids were fixed in 2% aqueous glutaraldehyde, stained with 2% aqueous uranyl acetate, and observed under the electron microscope.

### Surface staining of mRNA-transfected HEK293T and Vero E6 cells.

HEK293T and Vero E6 cells at 70%–80% confluence were individually transfected with 300 ng GP or 300 ng ANDV GPC, or were cotransfected with 300 ng GP and 100 ng VP40 mRNA in 24-well plates using the TransIT-mRNA Transfection Kit (Mirus Bio) following the manufacturer’s guidelines. Twelve hours and 24 hours after transfection, the cells were detached using a nonenzymatic cell dissociation solution (MilliporeSigma, C5914-100ML) and stained with a GP antibody cocktail of MR191, MR228, and MR235 (38), followed by staining with Alexa Flour 488 goat anti–human IgG (Invitrogen, Thermo Fisher Scientific, A11013). The cells were analyzed using the Accuri C6 Plus (BD Biosciences), and the data were analyzed using FlowJo software, version 10.9.0.

### Viruses.

The WT MARV strain Angola was used for PRNT assays, and the guinea pig–adapted MARV strain Angola was used for animal studies. The MARV strain 200501379 Angola was isolated during the outbreak in 2005 in Angola (65) and passaged 3 times in Vero-E6 cells. The guinea pig–adapted Angola strain of MARV used for infection of guinea pigs was provided by G. Kobinger (while at the Canadian National Microbiology Laboratory, Winnipeg, Manitoba, Canada). This virus was isolated originally from a patient in Angola, passaged once in Vero-E6 cells, passaged 8 times in Hartley guinea pigs using liver and spleen homogenates, once in Vero PP cells, and once in Vero E6 cells for stock production.

### Animal and biocontainment work.

All the work with infectious MARV was carried out in BSL-4 facilities at Galveston National Laboratory at UTMB. MARV-infected tissue and blood samples were inactivated by formalin and TRIzol for downstream processing following the UTMB Institutional Biosafety Committee–approved inactivation protocols. Similarly, plaque assay plates were also inactivated using an approved inactivation protocol.

### Guinea pig study 1.

Five-week-old female outbred Hartley guinea pigs were acquired from Charles River Laboratories. Guinea pigs were anesthetized with 5% isoflurane for vaccinations, blood collection, and virus challenge. On days 0 and 28, guinea pigs received prime and boost doses via the intramuscular route in the left leg (in 100 μL volume). Vaccines were diluted in PBS before administration. The vaccine doses were as follows: 10 μg GP mRNA (7.5 μg GP plus 2.5 μg NTFIX), 10 μg VLP mRNA (7.5 μg GP plus 2.5 μg VP40), 40 μg VLP mRNA (30 μg GP plus 10 μg VP40) and 40 μg NTFIX mRNA. Blood was collected on post-vaccination days 27 and 54. On day 56, guinea pigs were challenged i.p. with 1,000 PFU guinea pig–adapted MARV strain Angola. Post-challenge guinea pigs were monitored 1–3 times per day, depending on the disease score. Temperature, weight, and disease score were documented. Serum samples were collected after infection on days 3, 6, 9, and 12. Additionally, terminal serum and tissue samples (liver, spleen, and kidney) were collected on day 28 or when the guinea pig succumbed to infection.

### Guinea pig study 2.

Study 2 was conducted similarly to study 1 with the following changes. Guinea pigs received a booster dose on day 29. Post-prime vaccination blood was collected on day 29. The doses were as follows: 1, 3 and 7.5 μg GP mRNA, 1.33 μg VLP mRNA (1 μg GP plus 0.33 μg VP40), 4 μg VLP mRNA (3 μg GP plus 1 μg VP40), and 10 μg VLP mRNA (7.5 μg GP plus 2.5 μg VP40). The control group received PBS.

### Mouse study.

Six- to 7-week-old female BALB/c mice were acquired from Charles River Laboratories. Mice were anesthetized with 5% isoflurane for vaccinations and blood collection. On days 0 and 29, mice received prime and boost doses via the intramuscular route in the left leg (in 50 μL volume). Vaccines were diluted in PBS before administration. The doses were 1 μg GP mRNA and 1.33 μg VLP mRNA (GP 1 μg plus VP40 0.33 μg). On day 42, mice were euthanized, and their spleens were collected.

### Liver and spleen histopathology.

Organs obtained at necropsy were fixed in neutral buffered formalin and submitted to the institutional research histology service of the biorepository core for processing. Tissue cassettes were processed on a Sakura VIP6 tissue processor for routine histology (total run time of 28.5 hours). Embedding of tissue blocks was performed on an Epredia Histostar Embedding Station. Blocks were sectioned at 4 μm using the Thermo Fisher Scientific Microm-HM315 microtome. Finally, a standard staining protocol on the Sakura Tissue-Tek Prisma tissue stainer was used to stain the tissues with H&E.

### Bulk RNA-Seq and data analysis.

RNA sample quality was assessed using Agilent Bioanalyzer RNA Pico Chips (Agilent Technologies). RNA-Seq libraries were then prepared using the NEBNext PolyA module (New England Biolabs [NEB], 7490) and the Ultra II Directional RNA library preparation kit (NEB, 7760) following the manufacturer’s recommended procedure. The resulting libraries were run on Agilent Bioanalyzer High Sensitivity DNA Chips for size and quantified using qPCR. Sequencing was carried out on an Element Biosciences Aviti sequencer (Element Biosciences) using a paired-end 150 bp parameter to a sequencing depth of approximately 40 million paired reads per sample. The reads were quality filtered and trimmed for adapter sequence using Trimmomatic-0.39 (66) and aligned to the guinea pig reference genome Cavpor 3.0.112 using STAR 2.7.11a (67). Differential expression was assessed using the Bioconductor DESeq2 package (68), and functional enrichment analysis was performed using STRING, version 12.0 (69). Overlapping genes were analyzed using InteractiVenn (70).

Additional details on methods can be found in the Supplemental Methods.

### Data availability.

All source data values are provided in the Supporting Data Values file. RNA-Seq data were deposited in the NCBI’s Gene Expression Omnibus (GEO) database and (GEO GSE298124).

### Statistics.

Statistical analysis was performed using GraphPad Prism 10.2.3 (GraphPad Software). Data are presented as the median and IQRs or as individual values. The log-rank (Mantel-Cox) test was used to analyze survival data. Kruskal-Wallis analysis followed by Dunn’s multiple-comparison test or 2-way ANOVA followed by Dunn’s multiple-comparison test were used for group comparisons.

### Study approval.

All animal experiments were approved by the UTMB’s IACUC.

Author contributions. CS, AC, and AB conceived the study. CS, MM, SH, AC, and AB designed the study. CS, MM, MAH, MEC, HH, VLP, and HT performed experiments. CS, MM, HH, and HT analyzed the data. CS, MM, HH, HT, AC, and AB wrote the manuscript. JEC contributed reagents. AB supervised the study.

## Supplementary Material

Supplemental data

Supplemental data set 1

Supplemental data set 2

Unedited blot and gel images

Supporting data values

## Version 1 07/03/2025

In-Press Preview

## Version 2 09/02/2025

Electronic publication
