# Supplementary material for: Marburg virus glycoprotein mRNA vaccine is more protective than a virus-like particle-forming mRNA vaccine
Source: J Clin Invest. 2025 Jul 3;135(17):e194586. doi: 10.1172/JCI194586 (PMC12490202; doi:10.1172/JCI194586)
Supplement: Supplemental data [file jci-135-194586-s222.pdf]

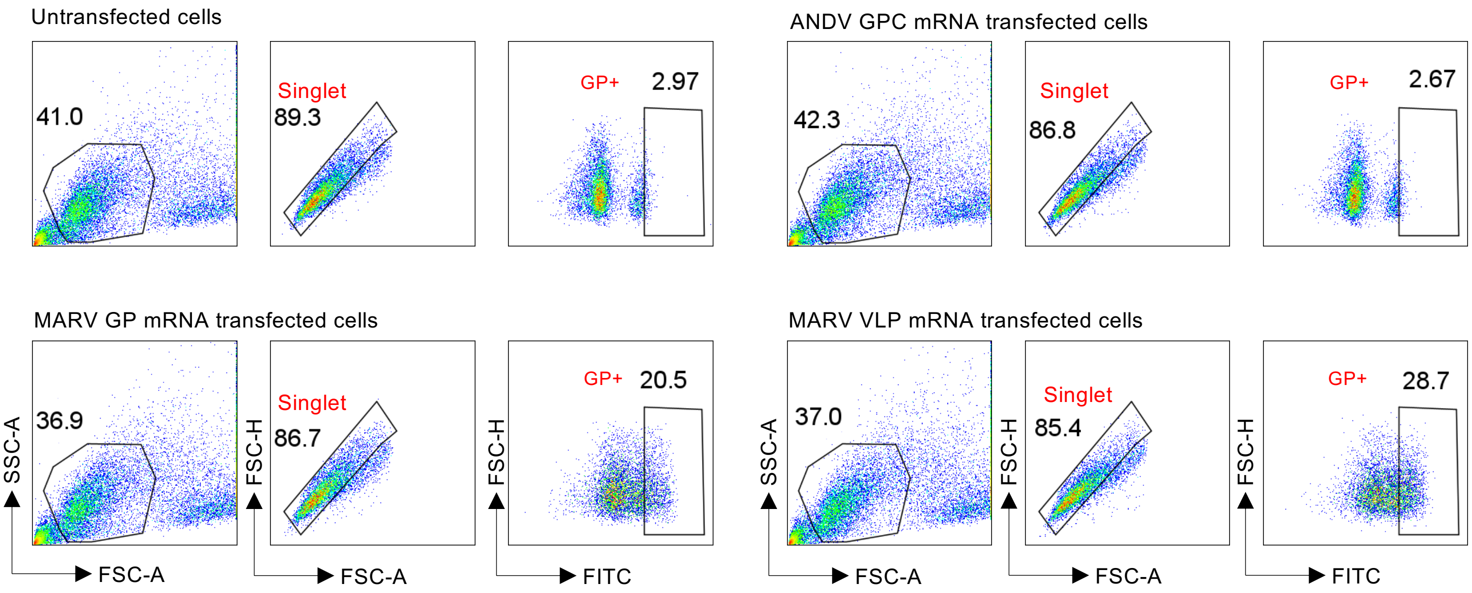

Supplemental Figure 1. Gating strategy and representative flow cytometry plots for surface GP staining. Percentages of the gated cell populations are indicated.

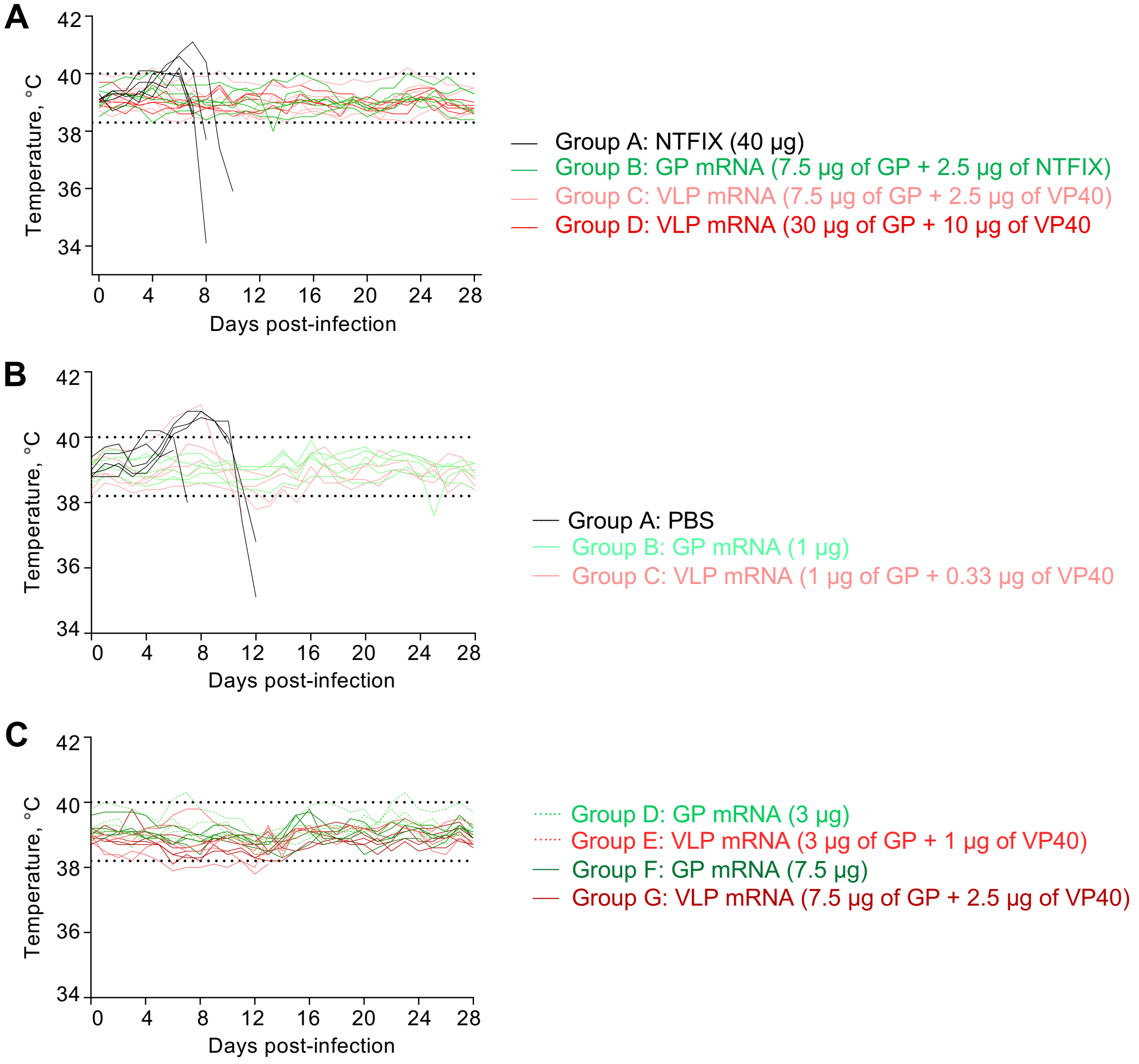

Supplemental Figure 2. Body temperatures of guinea pigs challenged with guinea pig-adapted MARV.
A. Body temperatures of guinea pigs from study 1.
B, C. Body temperatures of guinea pigs from study 2.
The dashed lines indicate the normal body temperature range. Data are represented as individual values.

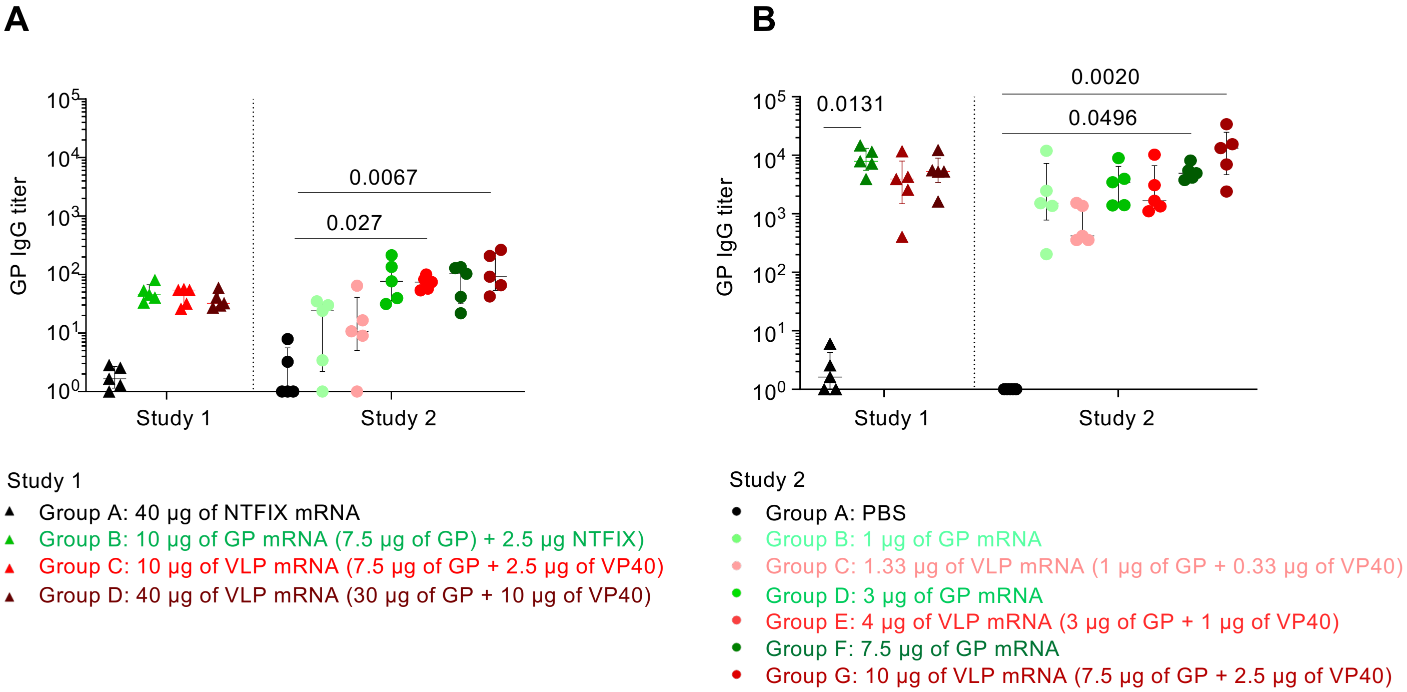

Supplemental Figure 3. MARV GP-specific binding IgG response: comparison of Studies 1 and 2.
A. Day 27 and 29 GP-specific IgG titers.
B. Day 54 GP-specific IgG titers.
Data are represented as medians and interquartile ranges. Statistical significance was calculated by Kruskal–Wallis analysis followed by Dunn's multiple comparison test.

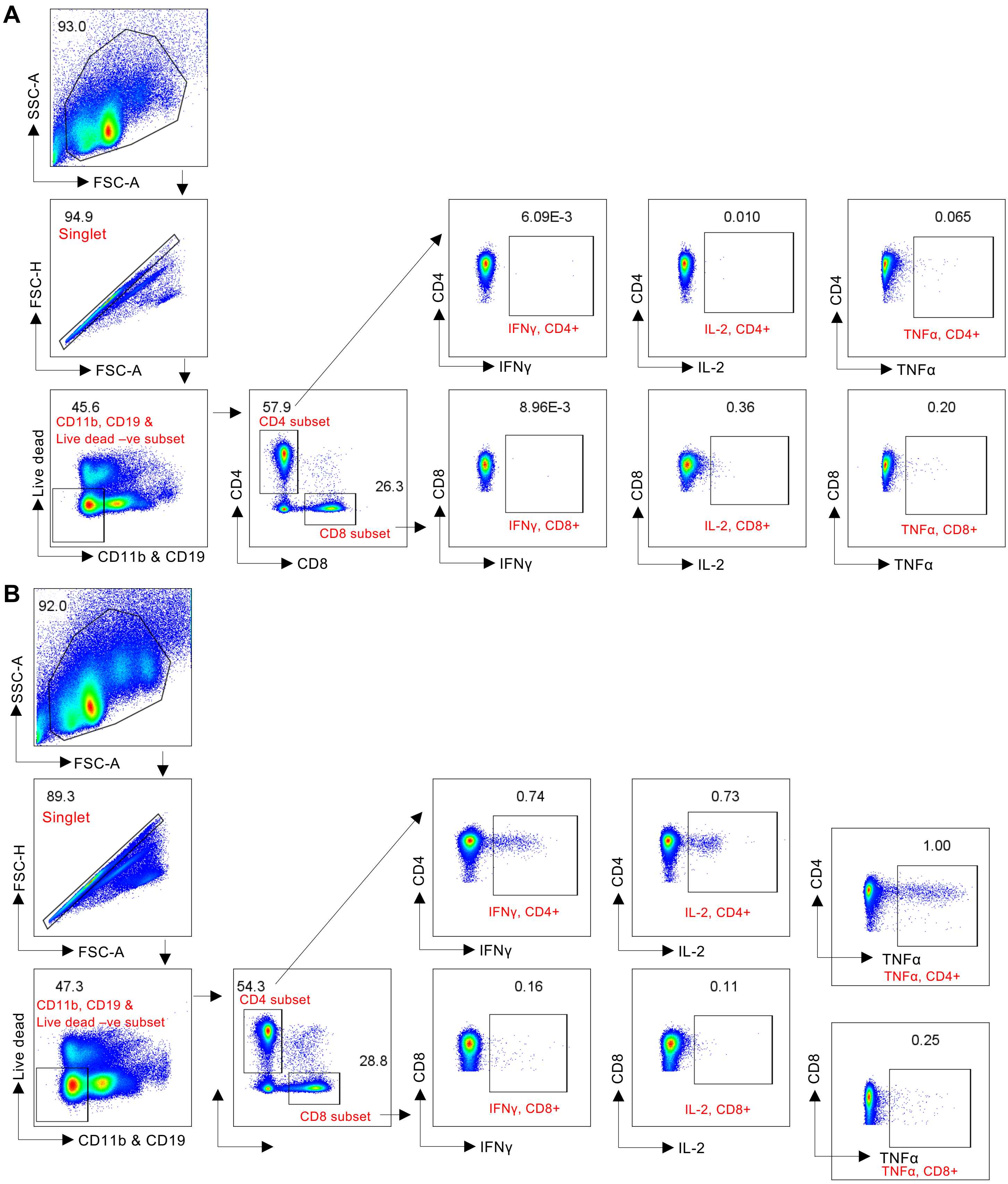

Supplemental Figure 4. Gating strategy and representative flow cytometry plots for T-cell assay.
Splenocytes from vaccinated BALB/c mice were stimulated with DMSO (control) or MARV GP-specific peptide pool to measure GP-specific $CD4^{+}$ and $CD8^{+}$ T cell responses by flow cytometry.
A. DMSO-treated cells (control).
B. GP-peptide pool treated cells.
Values indicate the percentages of the gated population.

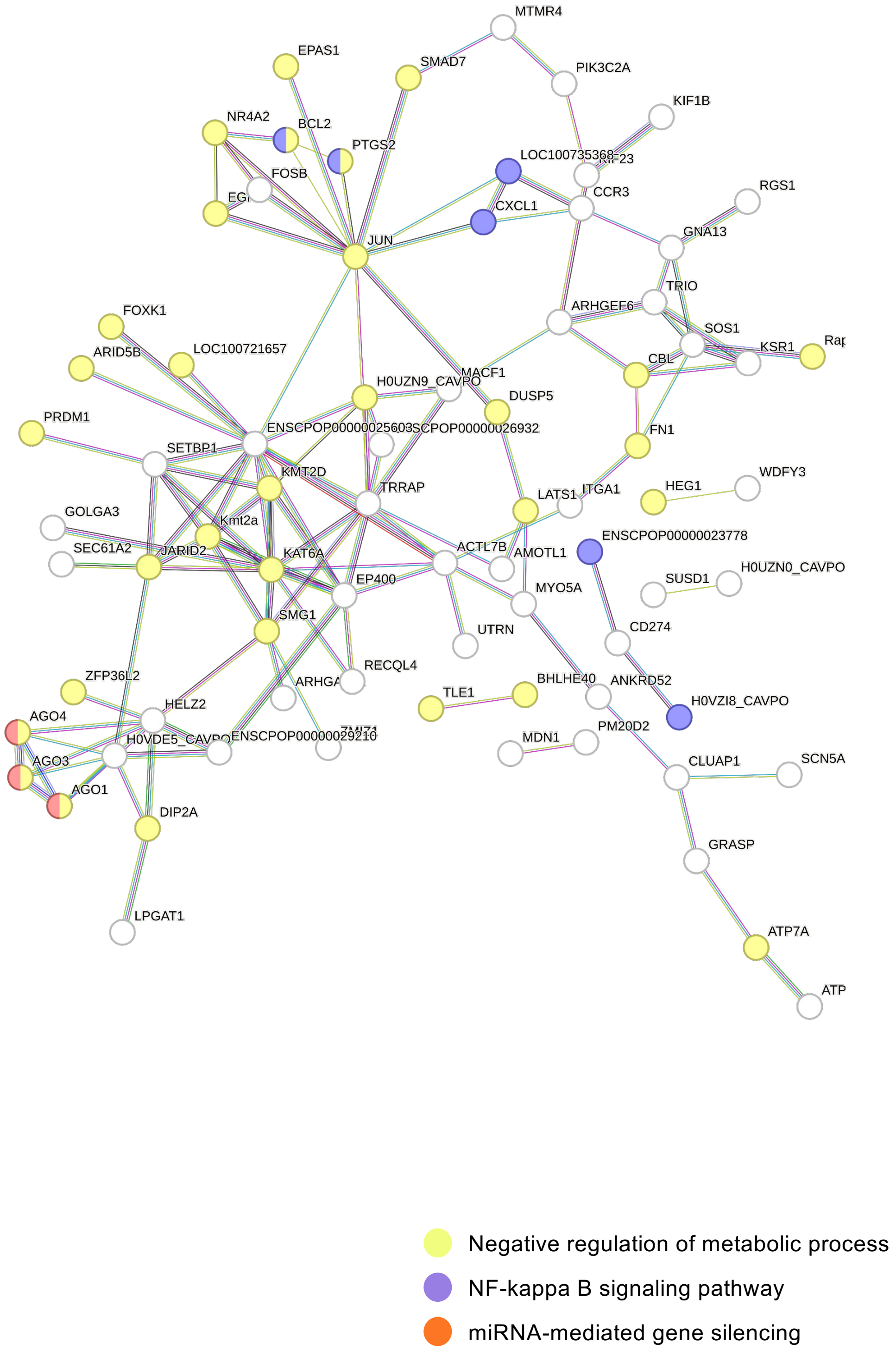

Supplemental Figure 5. Protein-protein interaction network among down-regulated genes in the VLP 10 μg vaccinated group.

Supplemental Table 1. Full Gene Names Corresponding to Gene Symbols Shown in Figures 10E and 11E.

**Figure 10E (Day 54 RNA-seq)**	**Gene Names**
	SIK1: Salt inducible kinase 1<br>HERC5: HECT and RLD domain containing E3 ubiquitin protein ligase<br>CCNDBP1: Cyclin D1 binding protein 1<br>IFIT1B: Interferon-induced protein with tetratricopeptide repeats 1B<br>RGS1: Regulator of G protein signaling 1 HMNC2: Hemicentin 2,<br>LOC100735514: Protein S100,<br>PDLIM4: Hemicentin 2,<br>PDLIM4: PDZ and LIM domain 4, NR4A2: nuclear receptor subfamily 4 group A member 2,<br>LOC100735368: C-C motif chemokine 4
**Figure 11E (Day 3 RNA-seq)**	TLR4: Toll Like Receptor 4<br>NLRC5: NLR Family CARD Domain Containing 5<br>IRF7: Interferon Regulatory Factor 7<br>IFIT1B: Interferon Induced Protein With Tetratricopeptide Repeats 1B<br>IFIT3: Interferon Induced Protein With Tetratricopeptide Repeats 3<br>IFIH1: Interferon Induced With Helicase C Domain 1<br>IFGGB1: Interferon-gamma-inducible GTPase<br>IFGGC3: nterferon-gamma-inducible GTPase<br>OAS1: DExD/H-box helicase 58<br>OAS3: 2'-5'-oligoadenylate synthetase 3<br>C1QC: Complement C1q C chain<br>C1QA: Complement C1q A chain<br>DDX58: DExH-box helicase 58<br>STING1: Transmembrane protein 173<br>EIF2AK2: Interferon-induced double-stranded RNA-activated protein kinase<br>TNFSF10: Tumor necrosis factor ligand superfamily membe<br>CASP10: Caspase 10

Supplemental Table 2. Summary of guinea pig study 1.

Vaccine	Dose	Survival	GP antibody titer		VP40 antibody titer		$PRNT_{60}$*	
			Day 27	Day 54	Day 27	Day 54	Day 27	Day 54
GP mRNA	7.5 µg	Survived	53	7887	N/A**	N/A	39	63
		Survived	33	3941	N/A	N/A	28	24
		Survived	45	14759	N/A	N/A	10	30
		Survived	40	11547	N/A	N/A	10	27
		Survived	81	7185	N/A	N/A	10	165
VLP mRNA	10 µg	Survived	57	11718	37	629	33	38
		Survived	32	3933	20	504	10	34
		Survived	26	2571	19	178	10	87
		Survived	54	4298	18	439	10	33
		Survived	54	405	7	12	10	10
VLP mRNA	40 µg	Survived	30	5294	6	81	10	48
		Survived	59	12323	316	37347	10	110
		Survived	27	1620	102	1309	10	55
		Survived	33	5236	143	2904	10	45
		Survived	41	5560	36	526	117	83

* For $PRNT_{60}$, the limit of detection is 10.

** N/A, not applicable, as the vaccine did not include VP40.

Supplemental Table 3. Summary of guinea pig study 2.

Vaccine	Dose	Survival or day of death*	Antibody titer GP		Antibody titer VP40		$PRNT_{60}$**	
			Day 27	Day 54	Day 27	Day 54	Day 27	Day 54
GP	1 µg	Survived	3	205	N/A*	N/A	10	10
		Survived	1	1518	N/A	N/A	10	10
		Survived	30	11955	N/A	N/A	10	19
		Survived	35	1363	N/A	N/A	10	10
		Survived	24	2481	N/A	N/A	10	10
VLP	1.33 µg	Survived	9	357	13	1	10	10
		Survived	17	356	9	5	10	10
		11	1	419	6	26	10	10
		Survived	65	1372	4	10	10	10
		Survived	11	1533	3	10	10	10
GP	3 µg	Survived	32	1406	N/A	N/A	10	10
		Survived	40	1394	N/A	N/A	10	10
		Survived	77	3969	N/A	N/A	10	17
		Survived	214	8919	N/A	N/A	10	10
		Survived	136	3478	N/A	N/A	48	161
VLP	4 µg	13	58	1664	1	32	10	17
		Survived	83	1346	5	218	10	20
		Survived	75	1105	3	21	10	10
		Survived	54	3086	9	142	10	20
		13	101	10247	2	97	10	10
GP	7.5 µg	Survived	103	8120	N/A	N/A	10	56
		Survived	134	3789	N/A	N/A	10	32
		Survived	129	5483	N/A	N/A	10	182
		Survived	42	4922	N/A	N/A	10	73
		Survived	22	4144	N/A	N/A	10	23
VLP	10 µg	Survived	208	6974	7	23	10	127
		Survived	66	13208	5	44	10	72
		Survived	93	15457	4	34	10	46
		Survived	265	33886	1	19	10	30
		Survived	42	2397	5	22	10	13

* Day after the vaccination.

** For $PRNT_{60}$, the limit of detection is 10.

*** N/A, not applicable, as the vaccine did not include VP40.

SUPPLEMENTAL METHODS

Analysis of T-cell response. The set of MARV GP-specific peptides was purchased from JPT Peptide Technologies (PepMix Marburgvirus (GP/Angola-05) and dissolved in DMSO according to the manufacturer's instructions. Mouse splenocytes were stimulated with MARV GP peptide mix (1 µg/ml) and CD28 monoclonal antibody (1 µg/ml) (Invitrogen, #14028182) for 6 h. The protein transport inhibitor brefeldin-A was added during the last 2 h of incubation (BD Biosciences, #555029). Following stimulation, cells were stained with LIVE/DEAD Fixable Aqua Dead Cell Stain Kit (ThermoFisher) and surface markers for CD19, CD11b, CD4 and CD8a (Biolegend Inc, PerCP/Cyanine5.5 anti-mouse CD19, #115534, PerCP/Cyanine5.5 anti-mouse/human CD11b, #101228, FITC anti-mouse CD4, #100510, APC/Cyanine7 anti-mouse CD8a, #100714) and then fixed with BD Cytofix/Cytoperm Fixation/Permeabilization Kit (BD Biosciences). Subsequently, intracellular staining (ICS) was performed with IFN-γ, TNF-α, and IL-2 (Biolegend Inc, Brilliant Violet 605 anti-mouse IFN-γ, #505840, PE anti-mouse TNF-α, #506306, APC anti-mouse IL-2, #503810) and analyzed using LSRFortessa (BD Biosciences). The data were analyzed using FlowJo v10.9.0.

MARV GP and VP40 IgG ELISA. Enzyme-linked immunosorbent assays (ELISAs) were conducted as described previously (1). Briefly, 8 ng/well MARV GPΔTM (IBT Bioservices, #0506-015) or 50 ng/well MARV VP40 (IBT Bioservices, #0568-001) were coated on 96-well plates (Greiner, #655061). Serum samples were tested in four-fold dilutions starting from 1:10 or 1:16 to 11 dilutions for GP antibody detection in duplicates. For VP40 antibody detection, serum samples were diluted four-fold, starting from 1:10 to 10 dilutions in duplicates. The remaining steps were carried out as described previously (1).

Assessment of MARV neutralizing antibodies and viremia. Both assays were performed as previously described (2). Briefly, serum samples were diluted twofold, starting from 1:10 to 11 dilutions in duplicates. The diluted serum was mixed with 200 PFU of MARV and incubated for 1 h at 37°C. The mixtures were then added to Vero E6 cell monolayers and incubated for 1 h at 37°C. Then, the virus/serum mixture was removed and incubated for 4 days in methylcellulose and minimal essential medium mixture. Finally, the cells were fixed in formalin and immunostained to visualize plaques. Viremia in serum samples was determined by incubating diluted serum samples in Vero E6 cell monolayers for 1 h at 37°C and then formalin fixation and

immunostaining. Viremia in tissue samples was also determined in Vero E6 cells by plaque assay using tissue homogenates.

REFERENCES

1. Kimble JB, Malherbe DC, Meyer M, Gunn BM, Karim MM, Ilinykh PA, et al. Antibody-Mediated Protective Mechanisms Induced by a Trivalent Parainfluenza Virus-Vectored Ebolavirus Vaccine. *J Virol.* 2019;93(4): e01845-18.
2. Meyer M, Garron T, Lubaki NM, Mire CE, Fenton KA, Klages C, et al. Aerosolized Ebola vaccine protects primates and elicits lung-resident T cell responses. *J Clin Invest.* 2015;125(8):3241-55.
