# Supplementary material for: Marburg virus glycoprotein mRNA vaccine is more protective than a virus-like particle-forming mRNA vaccine
Source: J Clin Invest. 2025 Jul 3;135(17):e194586. doi: 10.1172/JCI194586 (PMC12490202; doi:10.1172/JCI194586)
Supplement: Unedited blot and gel images [file jci-135-194586-s225.pdf]

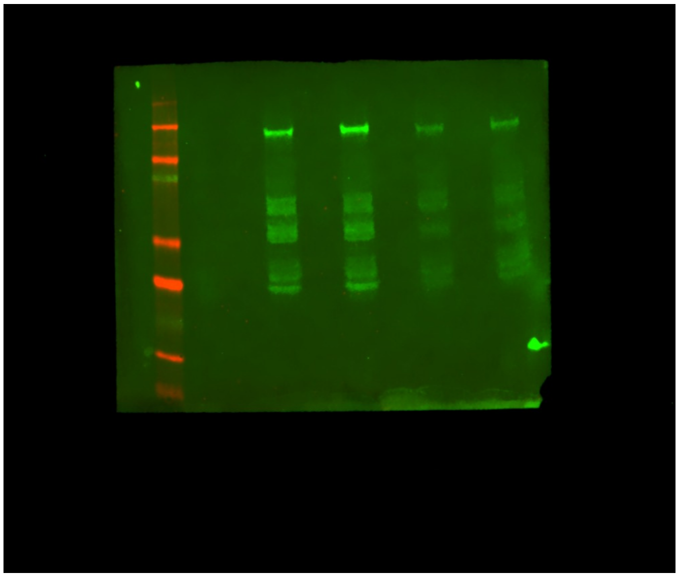

Fig. 1B. GP blot

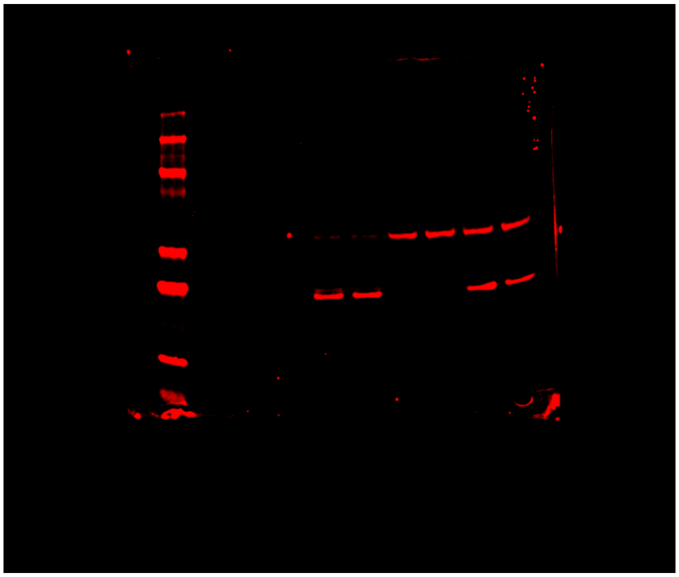

Fig. 1B. VP40 blot

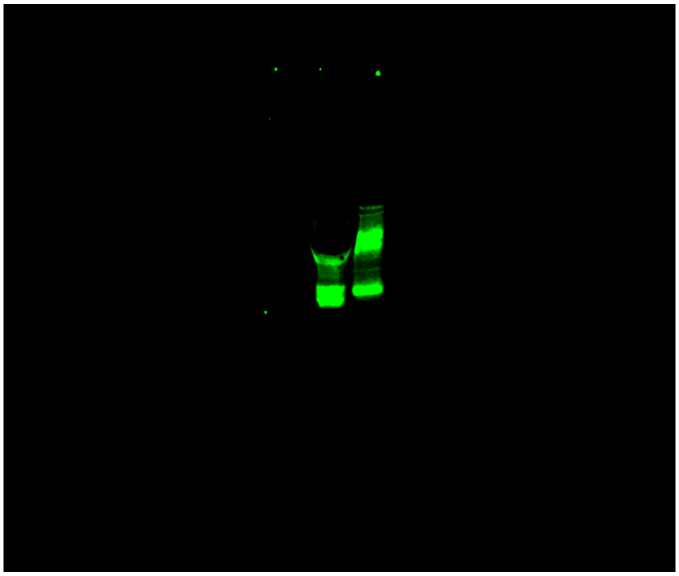

Fig. 1C. GP blot

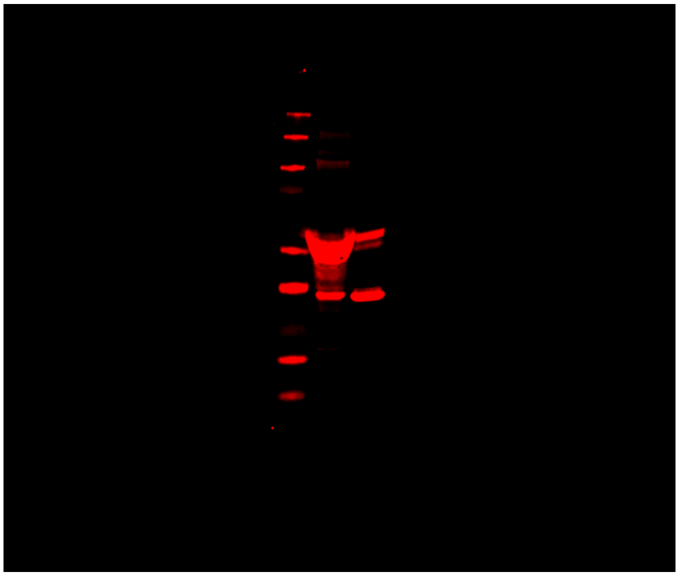

Fig. 1C. VP40 blot

Fig. 1. Optimization of MARV VLPs generation from GP and VP40 mRNA in HEK293T cells.

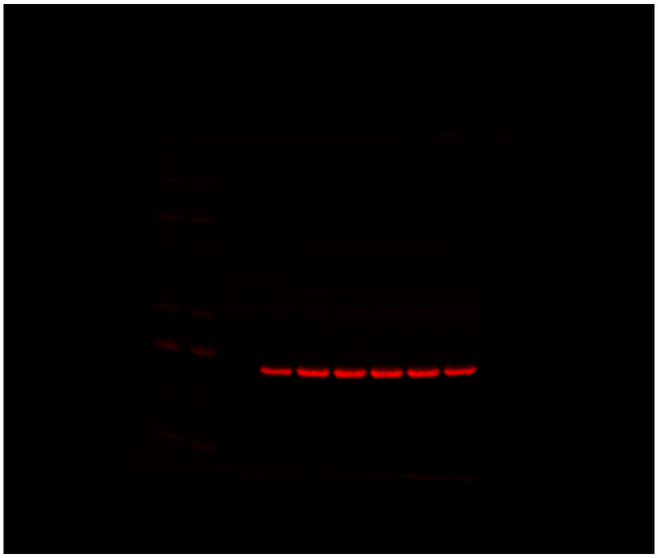

Fig. 1D. VP40 blot

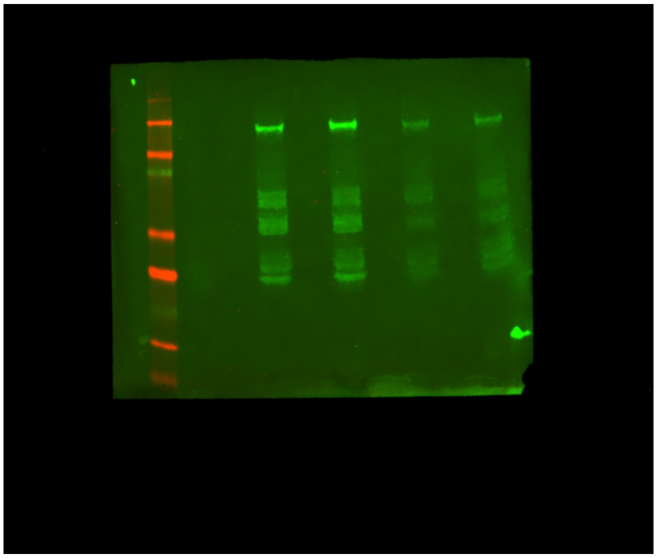

Fig. 1D. GP blot

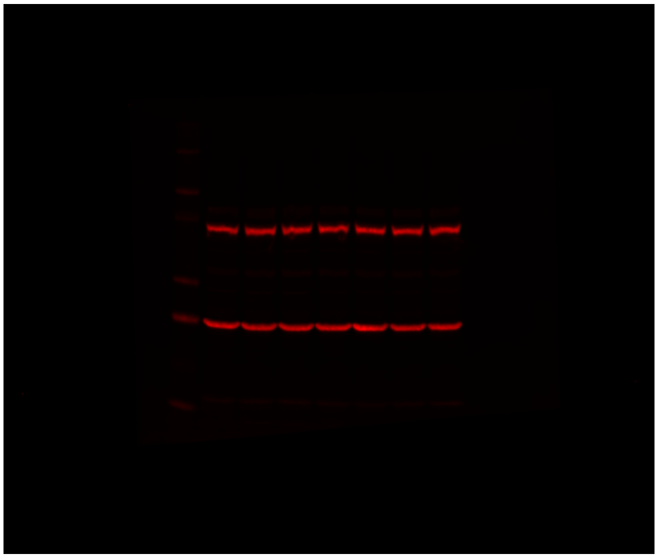

Fig. 1D. GAPDH blot

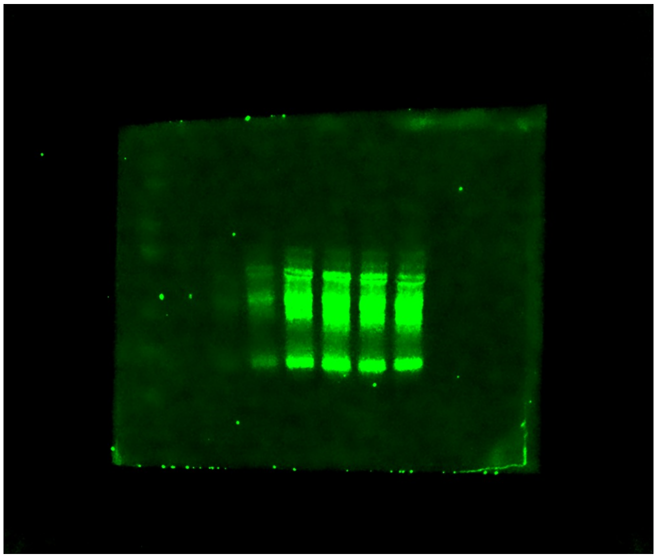

Fig. 1E. GP blot

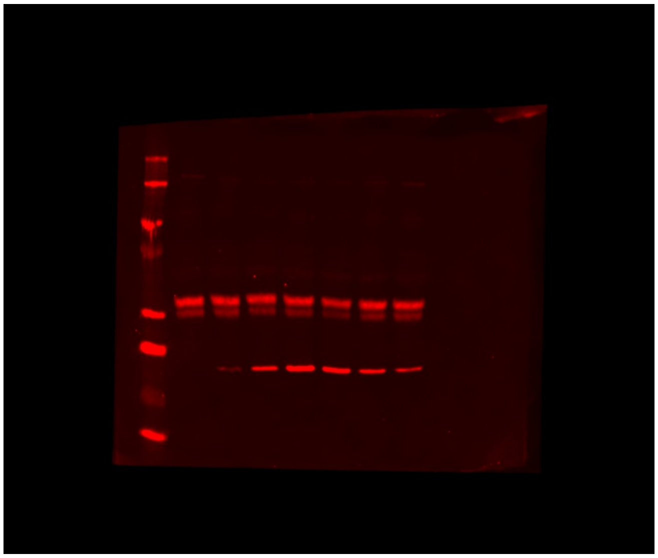

Fig. 1E. VP40 blot

Fig. 1. Optimization of MARV VLPs generation from GP and VP40 mRNA in HEK293T cells.
